# Knockout of *Vdac1* activates hypoxia-inducible factor through reactive oxygen species generation and induces tumor growth by promoting metabolic reprogramming and inflammation

**DOI:** 10.1186/s40170-015-0133-5

**Published:** 2015-08-26

**Authors:** M. Christiane Brahimi-Horn, Sandy Giuliano, Estelle Saland, Sandra Lacas-Gervais, Tatiana Sheiko, Joffrey Pelletier, Isabelle Bourget, Frédéric Bost, Chloé Féral, Etienne Boulter, Michel Tauc, Mircea Ivan, Barbara Garmy-Susini, Alexandra Popa, Bernard Mari, Jean-Emmanuel Sarry, William J. Craigen, Jacques Pouysségur, Nathalie M. Mazure

**Affiliations:** Institute for Research on Cancer and Aging of Nice, CNRS-UMR 7284-Inserm U1081, University of Nice Sophia-Antipolis, Centre Antoine Lacassagne, 33 Ave de Valombrose, 06189 Nice, France; Centre de Recherche en Cancérologie de Toulouse, INSERM–UPSIII U1037, Oncopole, Toulouse, 31037 Cedex 1 France; Centre Commun de Microscopie Appliquée, University of Nice Sophia-Antipolis, 28 Ave Valombrose, 06103 Nice, France; Department of Molecular and Human Genetics, Baylor College of Medicine, One Baylor Plaza, MS BCM225, Houston, TX 77030 USA; Institute for Research on Cancer and Aging of Nice, CNRS-UMR 7284-Inserm U1081, University of Nice Sophia-Antipolis, 28 Ave de Valombrose, 06107 cedex 02 Nice, France; INSERM U1065, Centre Méditerranéen de Médecine Moléculaire (C3M), Team Cellular and Molecular Physiopathology of Obesity and Diabetes, and University of Nice Sophia-Antipolis, Nice, France; Faculté de Médecine, LP2M - CNRS UMR-7370, Université de Nice Sophia Antipolis, 28 Avenue de Valombrose, Nice, 06107 cedex 2 France; Department of Microbiology and Immunology, Indiana University School of Medicine, Indianapolis, IN 46202 USA; Institute of Metabolic and Cardiovascular Diseases, INSERM U1048, Rangueil Hospital, 1 Avenue Professeur Jean Poulhes, BP 84225, 31432 Cedex 4 Toulouse, France; Institut de Pharmacologie Moléculaire et Cellulaire (IPMC), Centre National de la Recherche Scientifique, CNRS UMR 7275, Sophia Antipolis, & University of Nice Sophia-Antipolis, Nice, France; Centre Scientifique de Monaco (CSM), Monte Carlo, Sophia Antipolis, Monaco

## Abstract

**Background:**

Mitochondria are more than just the powerhouse of cells; they dictate if a cell dies or survives. Mitochondria are dynamic organelles that constantly undergo fusion and fission in response to environmental conditions. We showed previously that mitochondria of cells in a low oxygen environment (hypoxia) hyperfuse to form enlarged or highly interconnected networks with enhanced metabolic efficacy and resistance to apoptosis. Modifications to the appearance and metabolic capacity of mitochondria have been reported in cancer. However, the precise mechanisms regulating mitochondrial dynamics and metabolism in cancer are unknown. Since hypoxia plays a role in the generation of these abnormal mitochondria, we questioned if it modulates mitochondrial function. The mitochondrial outer-membrane voltage-dependent anion channel 1 (VDAC1) is at center stage in regulating metabolism and apoptosis. We demonstrated previously that VDAC1 was post-translationally C-terminal cleaved not only in various hypoxic cancer cells but also in tumor tissues of patients with lung adenocarcinomas. Cells with enlarged mitochondria and cleaved VDAC1 were also more resistant to chemotherapy-stimulated cell death than normoxic cancer cells.

**Results:**

Transcriptome analysis of mouse embryonic fibroblasts (MEF) knocked out for *Vdac1* highlighted alterations in not only cancer and inflammatory pathways but also in the activation of the hypoxia-inducible factor-1 (HIF-1) signaling pathway in normoxia. HIF-1α was stable in normoxia due to accumulation of reactive oxygen species (ROS), which decreased respiration and glycolysis and maintained basal apoptosis. However, in hypoxia, activation of extracellular signal-regulated kinase (ERK) in combination with maintenance of respiration and increased glycolysis counterbalanced the deleterious effects of enhanced ROS, thereby allowing *Vdac1*^*−/−*^ MEF to proliferate better than wild-type MEF in hypoxia. Allografts of RAS-transformed *Vdac1*^*−/−*^ MEF exhibited stabilization of both HIF-1α and HIF-2α, blood vessel destabilization, and a strong inflammatory response. Moreover, expression of *Cdkn2a*, a HIF-1-target and tumor suppressor gene, was markedly decreased. Consequently, RAS-transformed *Vdac1*^*−/−*^ MEF tumors grew faster than wild-type MEF tumors.

**Conclusions:**

Metabolic reprogramming in cancer cells may be regulated by VDAC1 through vascular destabilization and inflammation. These findings provide new perspectives into the understanding of VDAC1 in the function of mitochondria not only in cancer but also in inflammatory diseases.

**Electronic supplementary material:**

The online version of this article (doi:10.1186/s40170-015-0133-5) contains supplementary material, which is available to authorized users.

## Background

While the Warburg effect, or aerobic glycolysis, is considered to be primarily responsible for the metabolic reprogramming of cancer cells [1], mitochondrial respiration remains functional. However, it is not clear how mitochondria impact on proliferation or transformation of cancer cells, but as the «powerhouse» of cells, any change in metabolism can strongly influence the survival of the cancerous cell. Mitochondria are not only crucial in metabolic reprogramming; they also play an important role in delivering the message of cell death i.e., apoptosis. When the mitochondrial membrane potential (Δψm) is lost, mitochondria lose the integrity of their outer membrane, ATP synthesis is stopped, and proteins such as cytochrome C activate a cascade of caspases, ensuring certain death of the cell [2, 3].

The voltage-dependent anion channel (VDAC) is a major protein of the mitochondrial outer membrane that functions at the intersection of metabolism and apoptosis. The mammalian mitochondrial porin family includes three isoforms: VDAC1, VDAC2, and VDAC3 [4]. However, their expression levels differ according to the type of tissue, as do their physiological function. Mice lacking *Vdac1* or *Vdac3* are viable, whereas mice lacking *Vdac2* are not. While heterozygous *Vdac1*^+/−^ mice appear to have no obvious phenotype, when mated, they showed a reduced number of homozygous deficient mice when compared to the expected Mendelian ratio [5]. Only 40 % of the expected number of *Vdac1*^−/−^ mice survived, suggesting partial lethality, the reason for which has not yet been elucidated. VDAC1 is a pore that regulates the passage of molecules, including NADH, ATP/ADP, citrate, succinate, glutamate, pyruvate, and even glucose, as well as Mg^2+^, Ca^2+^, Cl^−^, K^+^, and Na^+^ ions [4]. It regulates the mitochondrial phenotype, apoptosis, and glycolysis through binding to hexokinase I/II (HKI/II), the first enzyme of the glycolytic pathway. Binding of HK to VDAC is a crucial event that coordinates mitochondrial ATP generation, cytoplasmic glycolytic flux, and possibly cytochrome C release. Moreover, HKI/II are upregulated by hypoxia-inducible factors (HIFs), the key transcription factors that direct the cell response to hypoxia, a condition in which tumors are inadequately oxygenated [6]. It is well known that the hypoxic nature of tumors is associated with poor clinical outcome, as hypoxia confers resistance to radio- and chemotherapy.

The role of VDAC1 in cancer has not been extensively investigated [7, 8, 4]. We recently reported the presence in tumor cells of a novel hypoxia-induced form of VDAC1 lacking the C-terminus (VDAC1-ΔC) [7, 9]. This new form was produced after long-term exposure to hypoxia. Cells expressing VDAC1-ΔC produced more ATP and were more resistant to stimulated apoptosis than cells expressing only full length VDAC1. In hypoxia, the level of full length VDAC1 decreased by about at least 50 % while the level of VDAC1-ΔC conversly increased by about 50 %. We hypothesized that knockout of VDAC1 in hypoxia would have a substantial impact on cell proliferation, oxidative and glycolytic metabolism, as well as apoptosis in hypoxic cells.

Herein, we characterized the cellular and molecular phenotype of *Vdac1*-deficient mouse embryonic fibroblasts (MEF) in normoxia or hypoxia. We report that VDAC1 participates in apoptosis and oxidative phosphorylation via reactive oxygen species (ROS) production, which modulates proliferation. We report connection to the Raf/MEK/ERK signaling pathway that counterbalances the deleterious effects of ROS in hypoxia. Finally, we show that knockout of *Vdac1* in MEF expressing oncogenic RAS potentiates tumor development in mice by promoting metabolic reprogramming, accelerating vascular destabilization and inflammation.

## Methods

### Cell culture, transfection, and animals

MEF cells were grown in Dulbecco’s modified eagle’s medium (DMEM) (Gibco-BRL) supplemented with 10 % fetal bovine serum with penicillin G (50 U/ml) and streptomycin sulfate (50 μg/ml). An INVIVO_2_ 200 anaerobic workstation (Ruskinn Technology Biotrace International Plc) set at 1 % oxygen, 94 % nitrogen, and 5 % carbon dioxide was used for hypoxic conditions. MEF were transformed with the pBabe-RAS^V12^ vector, and puromycin-resistant cells were collected. Animal procedures were approved by the Animal Care and Use Committee of the Unité Mixte de Service 006 of Toulouse (approval number 13-U1037-JES-08)*.*

### Electron microscopy

Cells were fixed in situ with 1.6 % glutaraldehyde in 0.1 M phosphate buffer at room temperature (RT) and stored overnight at 4 °C. Samples were rinsed in the same buffer and then postfixed with 1 % osmium tetroxide and 1 % potassium ferrocyanide in 0.1 M cacodylate buffer for 1 h at RT to enhance the staining of cytoplasmic membranes. Cells were rinsed with distilled water, embedded in epoxy resin, sectioned, and examined with a Philips CM12 transmission electron microscope equipped with an Olympus SIS CCD camera. The area of the mitochondria of cells was calculated as an ellipse (0.785ab; n.2, 50 mitochondria per experiment; mean ± SD).

### ATP determination

MEF (Wt and *Vdac1*^−/−^) and RAS MEF (Wt and *Vdac1*^−/−^) were incubated in hypoxia for 72 h and then lysed. Quantification of ATP was done using a luciferin/luciferase-based assay (Cell Titer Glo kit, Promega) according to the manufacturer’s instructions, and results are expressed as relative luminescence units (RLU). Each condition was tested eight times, and the entire experiment was done twice.

### Lactate measurement

The lactate concentration in the supernatant of cells incubated either in normoxia or hypoxia for 48 h was determined by an enzyme-based assay using 900 μM β-NAD (BioChemika) and 175 μg/ml l-lactate dehydrogenase (BioChemika), and 100 μg/ml glutamate-pyruvate transaminase (Roche) were diluted in a sodium carbonate (620 mM)-l-gultamate (79 mM) buffer adjusted to pH 10. Lithium lactate was used as a standard. Measurement was done with a microplate reader after incubation for 30 min at 37 °C. For each condition, the protein concentration was determined to express the lactate concentration as mmole/μg protein.

### Respirometry and extracellular acidification

The cellular oxygen consumption rate (OCR) and extracellular acidification rate (ECAR) were obtained using a Seahorse XF96 extracellular flux analyzer from Seahorse Bioscience (North Billerica, MA, USA). The final concentrations of the agents are given in the legends. Experiments were performed according to the manufacturer’s instructions. Protein standardization was performed after each experiment, with no noticeable differences in protein concentration and cell phenotype.

### Determination of glutamate production

Glutamate concentrations were measured in media supernatants using a Ysi 2300 STAT Plus analyzer (YSI Life Sciences). Measurements were done in triplicate, and the experiment was repeated two times.

### Caspase activation

Quantification of the caspase 3/7 activity was done using a luciferin/luciferase-based assay (Caspase-Glo 3/7 kit, Promega) according to the manufacturer’s instructions. Each condition was performed eight times, and the entire experiment was done three times. Significant differences are based on the Student’s *t* test (*p* < 0.005). STS was added 4 h prior to assay for caspase 3/7 activity.

### Flow cytometry and ROS measurement

For measuring intracellular cytosolic ROS levels, cells were cultured in hypoxia or normoxia for 48 h. Cells were then treated with oxidation sensitive DCF-DA (a final concentration of 10 μM) in culture medium for 30 min at 37 °C. After trypsination, the fluorescence of DCF in cells was measured using a fluorescence-activated cell sorter (BD FACSCalibur, analyzer). Mitochondrial H_2_O_2_ production was determined using Amplex UltraRed as described [10] but with a Xenius XC Safas spectrofluorimeter (Monaco) at λ_excitation_ = 560 nm, λ_emision_ = 590 nm. Whole digitonin permeabilized cells were used instead of isolated mitochondria, as described [11]. The assay was performed with 0.6 mg protein/ml of each cell type suspended in KHEP buffer without BSA (120 mM KCl, 2.5 mM MgCl_2_, 1 mM EGTA, 5 mM HEPES pH 7.2 at 37 °C) together with 5 U/ml horseradish peroxidase, 25 U/ml superoxide dismutase, and 50 μM Amplex UltraRed. The reaction was monitored and digitonin (final 10 μg/ml), then potassium succinate (final 5 mM), and then antimycin A (final 2 mM) were added successively. The results are expressed as the mean slope ratio of antimycin A/succinate.

### Immunoblotting

Cells were lysed in 1.5x SDS buffer and the protein concentration determined using the BCA assay. 40 μg of protein of whole cell extracts was resolved by SDS-PAGE and transferred onto a PVDF membrane (Millipore). Membranes were blocked in 5 % non-fat milk in TN buffer (50 mM Tris–HCl pH 7.4, 150 mM NaCl) and incubated in the presence of the primary and then secondary antibodies in 5 % non-fat milk in TN buffer.

The Bak antibody was purchased from Abcam, Bid and tBid from R&D Systems, Bax and Bcl-X_L_ from Santa Cruz, Bcl-2 from Novus, and Mcl-1 from Sigma. Rat anti-mouse CD31 (MEC 13.3) was from BD Bioscience (San Diego, CA). VDAC1 antibody (ab15895) was purchased from Abcam. Rabbit polyclonal anti-HIF-1α antibody (antiserum 2087) was produced and characterized in our laboratory [12]. ECL signals were normalized to either β-tubulin or ARD1 [13]. After washing in TN buffer containing 1 % Triton-X100 and then in TN buffer, immunoreactive bands were visualized with the ECL system (Amersham Biosciences).

### Microarray experiments

MEF were incubated in normoxia or hypoxia for 72 h (1 % O_2_) and then lysed prior to RNA isolation. RNA was assessed for integrity by using an Agilent BioAnalyser 2100 (Agilent Technologies) (RIN above 9). RNA samples were then labeled with Cy3 dye using the low RNA input QuickAmp kit (Agilent) as recommended by the supplier. Labeled cRNA probe (825 ng) was hybridized on 8x60K high density SurePrint G3 gene expression mouse microarrays. The experimental data are deposited on the NCBI Gene Expression Omnibus (GEO) under the series record number GSE63247 (http://www.ncbi.nlm.nih.gov/geo/query/acc.cgi?acc=GSE63247). Normalization of microarray data was performed using the Limma package available from Bioconductor (http://www.bioconductor.org). Inter-slide normalization was performed using quantile methods. Means of ratios from all comparisons were calculated and a B test analysis using paired analysis was performed. Differentially expressed genes were selected based on an adjusted *p* value below 0.01 and a log2 (fold change) >1. Data were analyzed for enrichment in biological themes (diseases and functions, canonical pathways, upstream analysis) using Ingenuity Pathway Analysis software (http://www.ingenuity.com/).

### Statistics

All values are the means ± SEM. Statistical analysis were performed using the Student’s *t* test as provided by Microsoft Excel. The *p* values are indicated. All categorical data used numbers and percentages. Quantitative data were presented using the median and range or mean. Differences between groups were evaluated using the chi-square test for categorical variables and the Student’s *t* test for continuous variables. Analyses were performed using SPSS 16.0 statistical software (SPSS Inc., Chicago, Ill). All statistical tests were two-sided, and *p* values <0.05 indicated statistical significance, whereas *p* values between 0.05 and 0.10 indicated a statistical tendency (Additional file 1).

### The online version of the article contains a data supplement

Additional file 2: Table S1, Additional file 3: Table S2, Additional file 4: Table S3, Additional file 5: Table S4, Additional file 6: Figure S1 and Additional file 7: Figure S2 show detailed data related to the microarray analysis. Additional file 8: Figure S3 shows expression of COX4-2. Additional file 9: Figure S4 shows the ROS status. Additional file 10: Figure S5 shows expression of GPX7 and the effect of ebselen. Additional file 11: Figure S6 shows changes in metabolic pathways. Additional file 12: Figure S7 shows data on glucose and glutamine catabolism. Additional file 13: Figure S8 shows the level of apoptosis. Additional file 14: Figure S9 shows electron micrograph and immunoblotting data related to autophagy. Additional file 15: Figure S11 shows data on vascularization of the RAS-MEF tumor tissue. Additional file 16: Figure S12 shows the immunohistochemistry to VEGF of tumor sections. Additional file 17: Figure S13 shows an *in silico* analysis of the *Vdac1* gene in cancer.

## Results

### High-throughput gene expression profiling of wild-type and *Vdac1*^*−/−*^ mouse embryonic fibroblasts

**Fig. 1:**
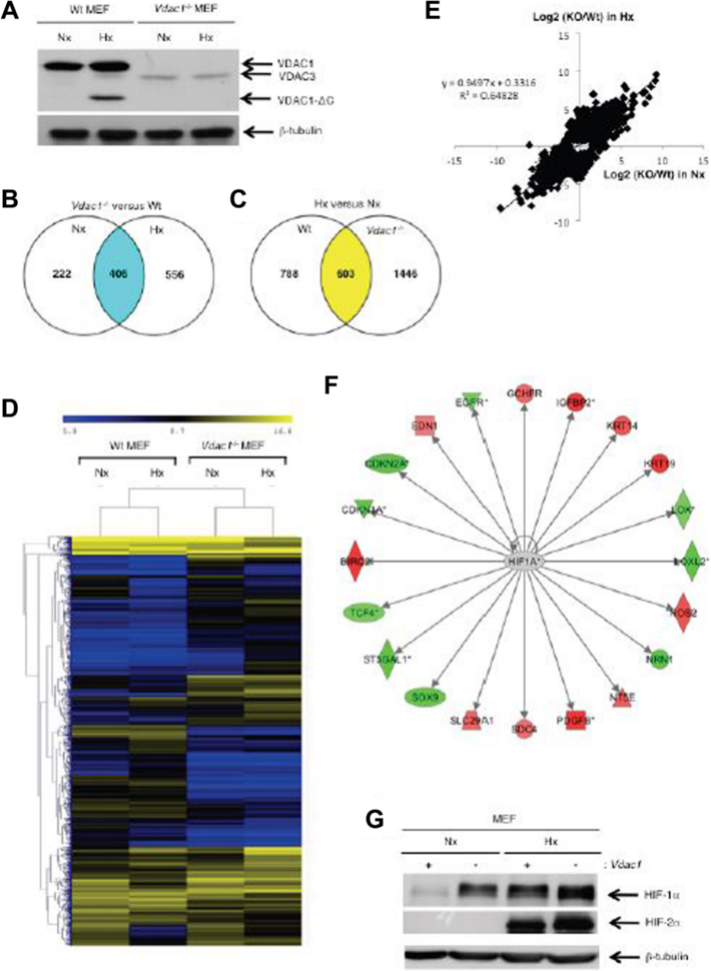
Characterization of wild-type (Wt) and VDAC1 null (*Vdac1* ^−/−^) mouse embryonic fibroblasts (MEF) in normoxia (Nx) and hypoxia 1 % O_2_ (Hx). **a** Wt and *Vdac1* ^−/−^ MEF were incubated in Nx or Hx for 72 h and cell lysates were analyzed by immunoblotting for VDAC. β-tubulin was used as a loading control. mRNA was extracted from Wt or *Vdac1* ^−/−^ cells after 72 h in Nx or Hx, and mRNA profiles were determined using pan-genomic microarrays. **b** Venn diagram depicting the numbers of genes significantly modulated between Wt vs *Vdac1* ^−/−^ MEF in normoxia (Nx) or hypoxia (Hx). **c** Venn diagram depicting the numbers of genes significantly modulated between Hx and Nx in Wt or *Vdac1* ^−/−^ MEF. **d** Heatmap comparing normalized log2 gene intensities of the top-ranked differentially expressed genes (1925 probes) for Wt and *Vdac1* ^−/−^MEF incubated in Nx or Hx (*n* = 2). The distance was measured using the Manhattan distance on the matrix of the log2 (intensity) and classification was performed using a complete agglomeration method. **e** Correlation between the change in the mRNA level between Wt and *Vdac1* ^−/−^MEF in normoxia and hypoxia. The determination coefficient (R^2^) was calculated using log2 fold changes from top modulated genes. **f** HIF-1α signaling pathway in normoxia in *Vdac1* ^−/−^ MEF vs Wt MEF. Red and green color codes for up- and down-regulation, respectively. **g** Wt (+) and *Vdac1* ^−/−^ (−) MEF were incubated in Nx or Hx for 72 h and cell lysates were analyzed by immunoblotting for HIF-1α and HIF-2α. β-tubulin was used as a loading control

We confirmed that VDAC-ΔC was produced in hypoxia in wild-type (Wt) MEF [14] and that the VDAC1 protein was not expressed in *Vdac1*^*−/−*^ MEF [5] (Fig. 1a). We then analyzed the transcriptome of Wt and *Vdac1*^*−/−*^ MEF in normoxia or hypoxia using a mouse whole genome microarray. Only RNA transcripts showing an adjusted *p* value <0.01 and an absolute log2 (fold change) >1 in at least one differential expression analysis were considered. Figure 1b and c recapitulates the number of genes differentially expressed when comparing the RNA transcript expression of *Vdac1*^*−/−*^ vs Wt MEF and the response of both cells lines to hypoxia, respectively. The data showed alteration in the gene expression profile of *Vdac1*^*−/−*^ MEF compared to Wt MEF in both normoxia and hypoxia (Fig. 1d, e). Analysis of the hypoxic response identified changes between the two cell lines (Fig. 1c, d). To understand the functional relevance of differentially expressed up- and down-regulated genes in *Vdac1*^*−/−*^ compared to Wt MEF, the microarray data were analyzed with Ingenuity Pathway Analysis^TM^ (IPA^TM^) software using the same cut-offs for both cells lines. The *Vdac1* deficiency, highlighted terms associated with «Diseases and functions» (Additional file 2: Table S1A), «Canonical pathways» (Additional file 2: Table S1B), and «Upstream regulators» (Additional file 2: Table S1C) in both normoxia and hypoxia. These results pointed to alterations in programs controlling cancer through metabolic pathways modulating HIF-1, cell death and survival, as well as cell proliferation and motility. Analysis revealed alterations in the hypoxic response of *Vdac1*^*−/−*^ MEF, including a lack of inhibition of cell proliferation, an increased oxidative stress response, and p53 activation (Additional file 3: Table S2). The «HIF-1α» signaling pathway both in normoxia (Fig. 1f) and hypoxia (Additional file 2: Figure S1) and the «Upstream regulators» «HIF-1α» were highlighted in *Vdac1*^*−/−*^ MEF (Additional file 2: Tables S1B-C and Additional file 16: S2B-C). The HIF-1α protein was detectable in normoxia in *Vdac1*^*−/−*^ MEF and increased in hypoxia to a level higher than that of Wt MEF in hypoxia, when equivalent numbers of cells were seeded (Fig. 1g). HIF-2α was also present at a higher level in *Vdac1*^*−/−*^ MEF, but only in hypoxia.

### *Vdac1*^*−/−*^ MEF proliferated faster than Wt MEF in hypoxia through activation of the ERK1/2 pathway

**Fig. 2:**
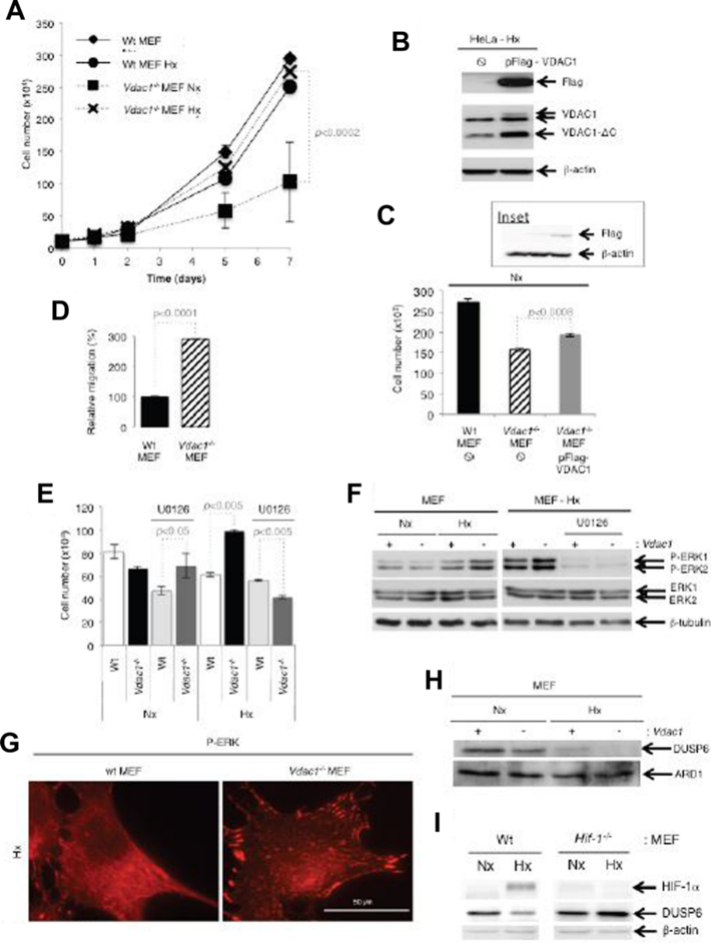
Characterization of the growth of Wt and *Vdac1* ^−/−^ MEF. **a** Both cell lines were seeded at the same density and incubated in Nx or Hx for the indicated number of days. The mean ± SEM is representative of four independent experiments carried out in duplicate. A *p* < 0.0002 shows significant difference from normoxia for the *Vdac1* ^−/−^ MEF. **b** Immunoblotting of HeLa cells transfected with pFlag-VDAC1. **c** Immunoblotting and proliferation assay of MEF tranfected with pFlag-VDAC1 **d** Relative migration of Wt and *Vdac1* ^−/−^ MEF in Nx as evaluated with a xCELLigence system. The mean ± SEM is representative of two independent experiments carried out in quadruplicate. **e** Characterization of the growth of Wt and *Vdac1* ^−/−^ MEF incubated in Nx or in Hx for 3 days in the absence or presence of an inhibitor of MEK (U0126, 10 μM). The mean ± SEM is representative of two independent experiments carried out in duplicate. **f** Wt (+) and *Vdac1* ^−/−^ (−) cells were incubated in Nx or Hx in the absence or presence of an inhibitor of MEK (U0126). Cell lysates were analyzed by immunoblotting for phospho-ERK (P-ERK), total ERK (ERK) and β-tubulin as a loading control. **g** Immunofluorescence of P-ERK in Wt and *Vdac1* ^−/−^ MEF in Hx. **h** Wt (+) and *Vdac1* ^−/−^ (−) MEF were incubated in Nx or Hx for 72 h and cell lysates were analyzed by immunoblotting for DUSP6. ARD1 was used as a loading control. (**i**) Immunoblotting of Wt MEF and Hif-1α^−/−^ MEF for DUSP6

The IPA^TM^ analysis highlighted cell proliferation and migration in *Vdac1*^*−/−*^ MEF in hypoxia with increased HIF-1 signaling (Additional file 3: Table S2A). Wt MEF showed exponential growth in normoxia but not in hypoxia (Fig. 2a). To examine if reintroduction of *Vdac1* into *Vdac1*^*−/−*^ MEF could reverse the effect on growth, we transiently transfected a pFlag-VDAC1 vector that has previously been shown to lead to expression of VDAC1 that integrates correctly into the mitochondrial membrane [15]. However, since MEF do not transfect easily, we first tested the vector on HeLa cells and confirmed both expression of VDAC1 and its post-translational cleavage in hypoxia (Fig. 2b), as we described [14, 9]. We observed that *Vdac1*^*−/−*^ MEF transfected with pFlag-VDAC1 proliferated slightly faster than empty vector transfected *Vdac1*^*−/−*^ MEF (Fig. 2c), suggesting that despite the low level of transfection, the absence of VDAC1 was responsible for the slow growth. Conversely, *Vdac1*^*−/−*^ MEF did not grow well in normoxia, but in hypoxia, showed similar growth to Wt MEF in normoxia. *Vdac1*^*−/−*^ MEF also migrated three times faster than Wt MEF in normoxia (Fig. 2d). To investigate whether the extracellular signal-regulated kinases 1 and 2 (ERK1/2) signaling pathway was involved, we cultured the cells in normoxia or hypoxia with or without U0126, a selective inhibitor of the intracellular Raf/MEK/ERK signaling pathway (Fig. 2e). In *Vdac1*^*−/−*^ MEF, U0126 had no effect on proliferation in normoxia, whereas proliferation in hypoxia was abolished with U0126 suggesting that the ERK1/2 pathway restored normal growth in hypoxia in *Vdac1*^*−/−*^ MEF. Expression of phospho-ERK1/2 was enhanced in hypoxia in *Vdac1*^*−/−*^ MEF compared to Wt MEF (Fig. 2e). P-ERK was predominantly localized in the cytoplasm of Wt MEF in hypoxia (Fig. 2g). However, active ERK targeted focal adhesion complexes in *Vdac1*^*−/−*^ MEF in hypoxia (Fig. 2g). The dual specificity phosphatase DUSP6, a mitogen-activated protein kinase phosphatases that dephosphorylates ERK, was highlighted in the IPA^TM^ analysis (Additional file 4: Table S3). A decrease in DUPS6 was observed in Wt MEF exposed to hypoxia and *Vdac1*^*−/−*^ MEF in normoxia (Fig. 2h). This decrease in expression was dependent on HIF-1 since the level of expression was the same in *Hif-1*^*−/−*^ MEF in normoxia or hypoxia (Fig. 2i). These results suggest that ERK activation allows cells to proliferate better in hypoxia through inactivation of DUSP6, but only in cells lacking *Vdac1*.

### *Vdac1*^*−/−*^ MEF showed alterations in mitochondrial respiration

**Fig. 3:**
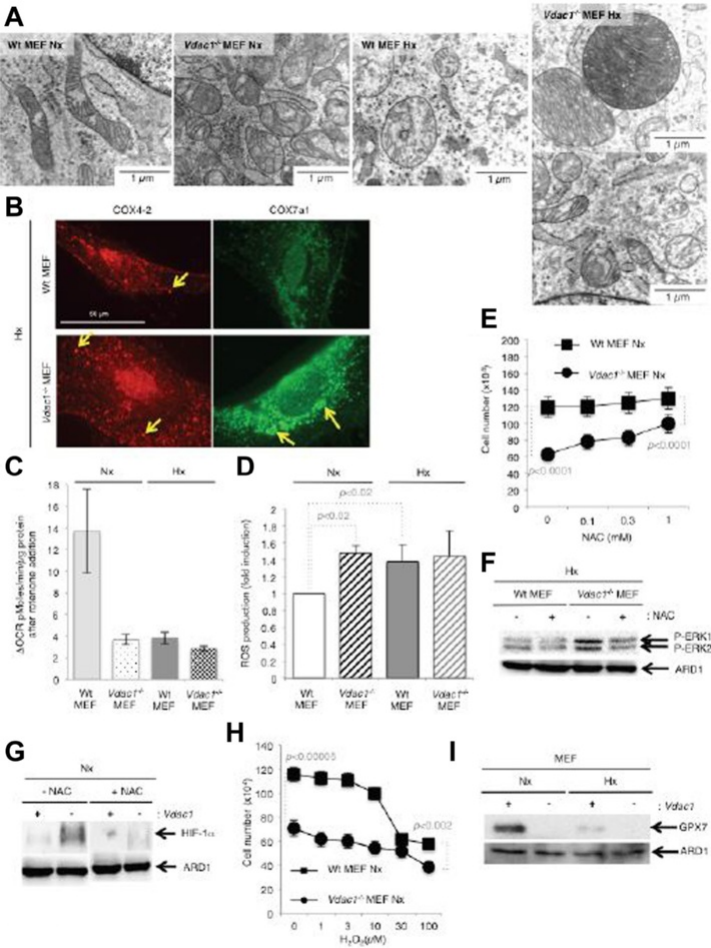
ROS production by Wt and *Vdac1* ^−/−^ MEF. **a** Representative electron micrographs of mitochondria of Wt and *Vdac1* ^−/−^ MEF incubated in normoxia (Nx) or hypoxia 1 % O_2_ (Hx) for 72 h. **b** Immunofluorescence to COX4-2 and COX7A1 in Wt and *Vdac1* ^−/−^ MEF in Hx for 72 h. **c** Respiratory control of Wt and *Vdac1* ^−/−^ MEF. Oxygen Consumption Rate (OCR) was measured in real time with a Seahorse XF bioenergetic system for Wt and *Vdac1* ^−/−^ MEF in Nx or Hx. ΔOCR was calculated from at least four measurements before and after treatment with rotenone at 1 μM. **d** Quantitative measurement of ROS production was done by staining with the fluorescent probe 2', 7'-dichlorofluorescin diacetate (DCFH-DA) followed by flow cytometry. These graphs are representative of four different experiments; *p* < 0.02, significant difference to Wt MEF in normoxia (Nx). **e** Wt and *Vdac1* ^−/−^ MEF seeded at the same density were incubated in Nx for 3 days in the presence of N-acetyl-l-cysteine (NAC, up to 1 mM). Mean ± SEM is representative of two independent experiments carried out in duplicate. **f** Wt and *Vdac1* ^−/−^ MEF were incubated in Hx for 72 h in the absence (−) or presence (+) of NAC (1 mM) and cell lysates were analyzed by immunoblotting for P-ERK. ARD1 was used as a loading control. **g** Wt (+) and *Vdac1* ^−/−^ (−) MEF were incubated in Nx for 24 h in the absence (−NAC) or presence (+NAC) of NAC (1 mM) and cell lysates were analyzed by immunoblotting for HIF-1α. ARD1 was used as a loading control. **h** Wt and *Vdac1* ^−/−^ MEF were incubated in Nx for 3 days in the presence of H_2_O_2_ (up to 100 μM). Mean ± SEM is representative of two independent experiments carried out in duplicate. **i** Wt (+) and *Vdac1* ^−/−^ (−) MEF were incubated in Nx or Hx for 72 h and cell lysates were analyzed by immunoblotting to GPX7. ARD1 was the loading control

As the IPA^TM^ analysis identified in hypoxic *Vdac1*^*−/−*^ MEF defects in expression of mRNAs implicated in mitochondrial respiration (Additional file 7: Figure S2), including complexes I, III, IV, and V, we hypothesized that these defects could generate ROS, which could then stabilize HIF-1α [16-18]. Mitochondria of Wt MEF in normoxia appeared as a tubular network with normal cristae (Fig. 3a), whereas hypoxic Wt MEF showed enlarged mitochondria with a modified organization of cristae, as reported [14]. The mitochondria of *Vdac1*^*−/−*^ MEF were enlarged, but in both normoxia and hypoxia. In addition, hypoxic mitochondria of *Vdac1*^*−/−*^ MEF showed two different populations of mitochondria. One (around 50 %; data not shown) exhibited enlarged mitochondria with a high number of cristae, as for tissues with a high energy demand [19], while the other population had the same morphology as normoxic mitochondria of *Vdac1*^*−/−*^ MEF. These results suggested that *Vdac1*^*−/−*^ MEF had a higher rate of respiration in hypoxia than in normoxia, possibly due to more cristae, which could promote proliferation. We quantified mitochondrial respiration with the Seahorse XF by measuring the OCR. Table 1 summarizes the mitochondrial coupling and respiratory control of MEF, as previously described [20]. Basal respiration (a) of normoxic *Vdac1*^*−/−*^ MEF was lower than in Wt MEF (*p* < 0.005). In hypoxia, the basal respiration of Wt MEF was substantially decreased (80 %) while that of *Vdac1*^*−/−*^ MEF was reduced by only 35 %. In normoxia and hypoxia, Wt and *Vdac1*^*−/−*^ MEF had a similar rate of proton leak (b), suggesting that mitochondria are not damaged, consistent with the morphology of the mitochondria. The rate of mitochondrial ATP synthesis (c) was also lower in *Vdac1*^*−/−*^ MEF in normoxia; however, although the ATP turnover was strongly inhibited in both cell lines in hypoxia, the ATP turnover of *Vdac1*^*−/−*^ MEF remained two-fold higher than that of Wt MEF. In addition, maximal respiration (d) was lower in *Vdac1*^*−/−*^ MEF in normoxia, but was greater or equal to that of Wt MEF in hypoxia. The coupling efficiency (c/a) was twofold lower in hypoxia than in normoxia in Wt and *Vdac1*^*−/−*^ MEF. The respiratory control ratio (d/b) was systematically decreased in *Vdac1*^*−/−*^ MEF. These results confirmed that: (i) normoxic mitochondria of *Vdac1*^*−/−*^ MEF exhibited substantial defects in respiratory control and that (ii) hypoxic mitochondria of *Vdac1*^*−/−*^ MEF were more efficient than hypoxic Wt MEF. However, the spare respiratory capacity (d-a) indicated that the hypoxic mitochondria had reached their bioenergetic limit. To explain the abundance of cristae and higher respiration in hypoxia in *Vdac1*^*−/−*^ MEF, we investigated the expression of cytochrome oxidase (COX). The level of COX4-1, was slightly decreased in hypoxia in both cell lines (data not shown), whereas COX4-2 showed a marked increase in hypoxia in both cell lines (Fig. 3b), as described [21]. A higher level of COX4-2 and COX7A1 (more dots) in *Vdac1*^*−/−*^ MEF in hypoxia than in Wt MEF was detected (Fig. 3b, Additional file 8: Figure S3A). Blockade of COX4-2 expression and of proliferation of *Vdac1*^*−/−*^ MEF in hypoxia in the presence of PD184352, a specific inhibitor of MEK, suggested that COX4-2 could regulate proliferation in hypoxia through the activation of ERK (Additional file 8: Figure S3B). However, while silencing of COX4-2 and COX7A1 with siRNA diminished substantially the respective mRNA levels, only silencing of COX7A1 decreased proliferation in *Vdac1*^*−/−*^ MEF without modifying apoptosis (data not shown).

**Table 1:** Respiratory control of Wt and *Vdac1* ^−/−^ MEF

	Nx		Hx	
	Wt MEF	*Vdac1* ^−/−^ MEF	Wt MEF	*Vdac1* ^−/−^ MEF
**(a)** Basal respiration	20.52 ± 2.5	16.19 ± 1.8	4.5 ± 0.52	10.4 ± 2.41
**(b)** Oligomycin-insensitve respiration (leak)	5.96 ± 0.9	5.04 ± 0.9	2.4 ± 0.27	7.1 ± 2.73
**(c)** Oligomycin-sensitive (ATP turnover) respiration	14.56 ± 1.6	11.16 ± 0.8	1.8 ± 0.70	3.2 ± 0.95
**(d)** Maximal respiration in the presence of FCCP	29.21 ± 3.2	19.29 ± 1.7	5.4 ± 1.19	6.0 ± 0.29
**(c/a)** Coupling efficiency	0.71 ± 0.02	0.71 ± 0.01	0.4 ± 0.12	0.3 ± 0.13
**(d/b)** Respiratory control ratio	4.91 ± 0.2	3.95 ± 0.4	2.3 ± 0.71	0.8 ± 0.44
**(d-a)** Spare respiratory capacity	8.19 ± 1.2	3.10 ± 0.1	1.3 ± 0.50	−5.4 ± 2.83


The oxygen consumption rate (OCR) was measured in real time with a Seahorse XF bioenergetic system for Wt and *Vdac1* ^−/−^MEF in Nx or Hx. The average OCR was calculated from at least four measurements during treatment with each compound (oligomycin, FCCP, rotenone/antimycin A) at a 1 μM final concentration.

(a) represents basal respiration, (b) oligomycin-insensitive respiration, (c) oligomycin-sensitive respiration, and (d) maximal respiration in the presence of FCCP. The graph shows how to define a, b, c, and d. The mean ± SEM is representative of six independent experiments carried out in quadruplicate

Blockade of complex I with rotenone in Wt MEF markedly decreased basal respiration, suggesting a normal complex I activity in normoxia (Fig. 3c). Inhibition with rotenone did not affect complex I activity in hypoxia as it is known that its activity is already reduced in hypoxia [22]. However, rotenone did not modify the OCR of *Vdac1*^*−/−*^ MEF, which suggests that the complex I activity was already diminished. We then quantified the levels of ROS (Fig. 3d). We found a strong correlation between the inactivation of complex I and ROS production. In addition, using a cuvette-based Amplex UltraRed assay for mitochondrial production of H_2_O_2_, *Vdac1*^*−/−*^ MEF mitochondria produced more H_2_O_2_ than did the Wt MEF (Additional file 9: Figure S4A). N-acetyl-l-cysteine restored proliferation of *Vdac1*^*−/−*^ MEF in normoxia to a similar level to that of Wt MEF (Fig. 3e), decreased P-ERK in hypoxia (Fig. 3f), and decreased HIF-1α in normoxia (Fig. 3g). Addition of hydrogen peroxide to Wt MEF decreased proliferation to a level similar to that of *Vdac1*^*−/−*^ MEF (Fig. 3h). The expression of enzymes involved in upstream or downstream transformation of H_2_O_2_ including superoxide dismutase 3 (*Sod3*) and glutathione peroxidase 7 (*Gpx7*) were tightly regulated in *Vdac1*^*−/−*^ MEF (Additional file 5: Table S4). However, SOD1, SOD2, and SOD3 showed no difference in expression in normoxia (Additional file 10: Figure S5B) or hypoxia (data not shown) in Wt MEF compared to *Vdac1*^*−/−*^ MEF. GPX7, which detoxifies hydroperoxide substrates, was absent in *Vdac1*^*−/−*^ MEF in both normoxia and hypoxia (Fig. 3i, Additional file 10: Figure S5A). Moreover, ebselen, a mimetic of GPX [23], did not modify HIF-1α stability (Additional file 10: Figure S5B). It decreased slightly the proliferation of Wt MEF, but not *Vdac1*^*−/−*^ MEF (Additional file 10: Figure S5C) and increased slightly the viability of *Vdac1*^*−/−*^ MEF (Additional file 10: Figure S5D), suggesting a putative protection against ROS in these cells.

These results showed that the accumulation of ROS in *Vdac1*^*−/−*^ MEF, probably due to down-regulation of GPX7 and the stabilization of HIF-1α, participate in the blockade of proliferation in normoxia. However, in hypoxia, activation of P-ERK and induced expression of COX4-2 and COX7A1 compensated for the reduced mitochondrial complex I activity in *Vdac1*^*−/−*^ MEF.

### *Vdac1*^*−/−*^ MEF were more glycolytic in hypoxia

**Fig. 4:**
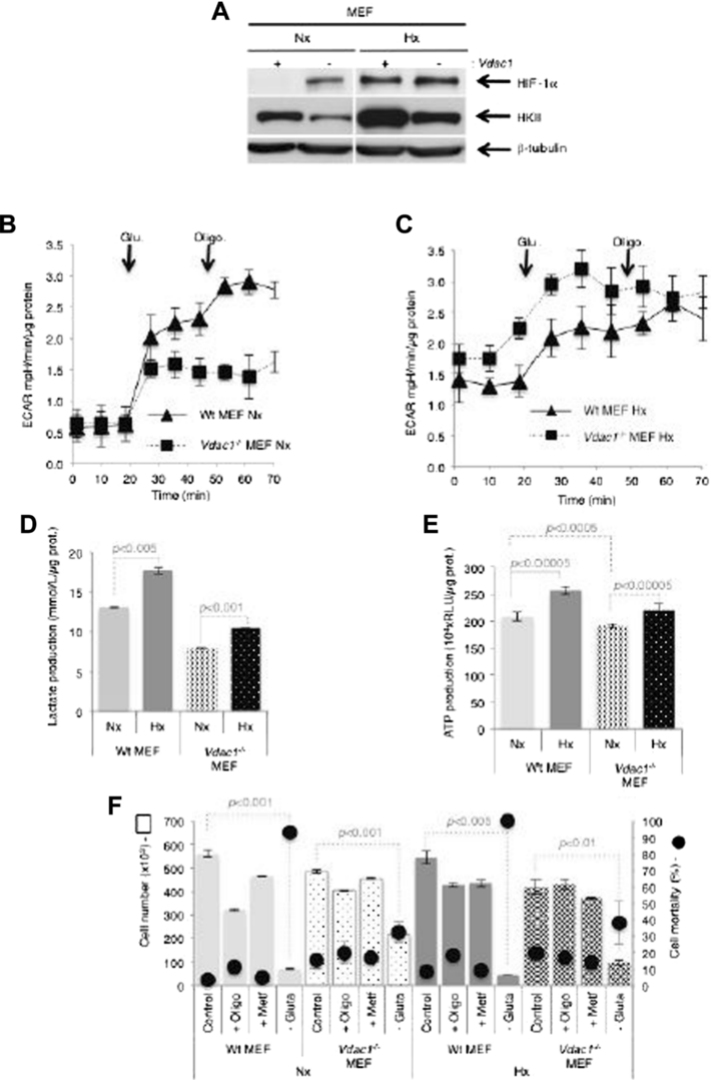
Metabolic characteristics of Wt and *Vdac1* ^−/−^ MEF incubated in normoxia (Nx) or hypoxia 1 % O_2_ (Hx). **a** Wt (+) and *Vdac1* ^−/−^ (−) MEF were incubated in Nx or Hx for 72 h and cell lysates were analyzed by immunoblotting for HIF-1α and HKII. β-tubulin was used as a loading control. The extracellular acidification rate (ECAR) in **b** Nx or **c** Hx of Wt and *Vdac1* ^−/−^ MEF was evaluated with a Seahorse XF bioenergetic system. Cells were deprived of glucose for 1 h, then glucose (Glu 10 mM) and oligomycin (Oligo 1 μM) were injected at the indicated times. **d** After 3 days of culture, cells were lysed in Assay Buffer with sonication. The amount of lactate was quantified in cell extracts. The mean ± SEM is representative of three independent experiments carried out in duplicate. A *p* < 0.001 and *p* < 0.005 show significant differences. **e** ATP production of Wt and *Vdac1* ^−/−^ MEF in Nx or Hx for 72 h. The mean ± SEM is representative of three independent experiments carried out in duplicate. A *p* < 0.0005 and *p* < 0.00005 show significant differences. **f** Wt and *Vdac1* ^−/−^ MEF were cultured for 2 days in Nx or Hx in the presence of oligomycin (Oligo, 1 μM), metformin (−Metf), and without glutamine (−Gluta), in the presence of dialyzed serum. The cell number was measured on a Beckman Coulter apparatus (*squares*). The percentage cell mortality was measured by trypan blue exclusion (*black dots*). The mean ± SEM is representative of two independent experiments carried out in duplicate. A *p* < 0.01, *p* < 0.001, and *p* < 0.005 show significant differences

IPA^TM^ analysis revealed that metabolism was minimally modified in *Vdac1*^*−/−*^ MEF compared to Wt MEF (Additional file 11: Figure S6). Nonetheless, hypoxic induction of HKII was maintained in both cell lines, but the basal amount of HKII in normoxia was lower in *Vdac1*^*−/−*^ MEF (Fig. 4a). We used the Seahorse XF to quantify glycolysis by measuring the ECAR, which primarily reflects lactate. Addition of oligomycin, which blocks mitochondrial ATP production, showed that the glycolytic capacity of *Vdac1*^*−/−*^ MEF was more than twofold lower than that of Wt MEF in normoxia (0.94 vs 2.34, respectively) (Fig. 4b). However, this ratio changed in hypoxia since (i) the basal level of glycolysis (1.75 vs 1.42) and (ii) the maximum capacity (1.45 vs 1.3) of *Vdac1*^*−/−*^ MEF was greater than that of Wt MEF (Fig. 4c). Relative to normoxia, both lactate and ATP production were increased in *Vdac1*^*−/−*^ and Wt MEF in hypoxia (Fig. 4d, e). *Vdac1*^*−/−*^ MEF were less sensitive to blockade of respiration (oligomycin or metformin) (Fig. 4f). As Wt MEF were extremely sensitive to the absence of glutamine (almost 100 % cell death in normoxia and hypoxia), the cell death of *Vdac1*^*−/−*^ MEF was about 32.3 % in normoxia and 38.1 % in hypoxia, suggesting an important role of glutamine, which is metabolized to glutamate in mitochondria, in *Vdac1*^*−/−*^ MEF. In the presence of 2-deoxy-d-glucose, a glucose analog that inhibits glycolysis, or in the absence of glucose, *Vdac1*^*−/−*^ and Wt MEF showed similar survival (Additional file 12: Figure S7A). Finally, *Vdac1*^*−/−*^ MEF produced little or no glutamate in both normoxia and hypoxia compared to Wt MEF (Additional file 12: Figure S7B). These results confirm that the *Vdac1*^*−/−*^ MEF grew better in hypoxia, by maintaining respiration and promoting glycolysis.

### *Vdac1*^*−/−*^ MEF showed a higher level of apoptosis than Wt MEF

**Fig. 5:**
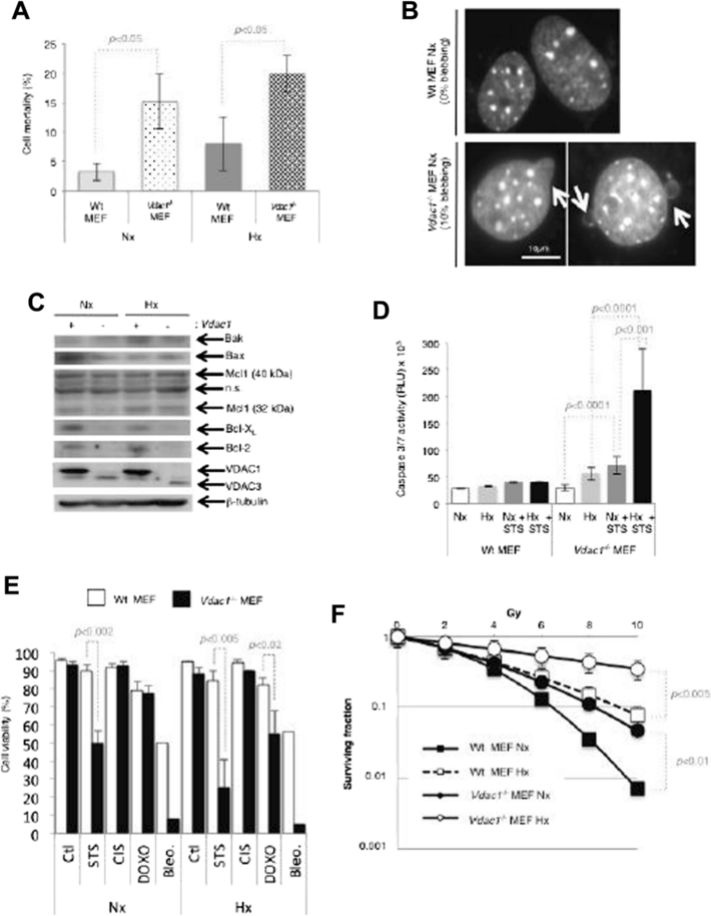
Knockout of *Vdac1* maintains a basal level of apoptosis. **a** Wt and *Vdac1* ^−/−^ MEF were cultured for 3 days in normoxia (Nx) or hypoxia 1 % O_2_ (Hx). The percentage cell mortality was measured by trypan blue exclusion. A *p* < 0.05 shows a tendency from the basal apoptosis of Wt MEF. **b** Cells were stained with DAPI (blue) to highlight the nucleus and its morphology. Quantification of the percentage of blebbing in Wt and *Vdac1* ^−/−^ MEF. At least 200 nuclei were counted blindly. **c** Wt (+) and *Vdac1* ^−/−^ (−) MEF were incubated in Nx or Hx for 72 h and cell lysates were analyzed by immunoblotting for Bak, Bax, Mcl-1, Bcl-X_L_, Bcl-2, VDAC, and HKII. β-actin was used as a loading control. **d** Wt and *Vdac1* ^−/−^ MEF were incubated in Nx or Hx for 72 h and challenged with staurosporin (STS) (1 μM) for 4 h. Apoptosis was evaluated from the level of caspase 3/7. A *p* < 0.001 and *p* < 0.0001 show significant differences. **e** Wt and *Vdac1* ^−/−^ MEF were cultured for 2 days and then treated for 3 days with staurosporine (STS) (1 μM), cisplatin (CIS) (2 μg/ml), doxorubicin (DOXO) (4 μg/ml), or bleomycin (Bleo) (10 μg/ml). Cell viability was measured using an ADAM cell counter. A *p* < 0.02, *p* < 0.002, and *p* < 0.005 show significant differences. **f** Radioresistance of Wt and *Vdac1* ^−/−^ MEF cultured for 24 h in Nx or Hx and treated with the indicated dose of radiation. Cell growth was then evaluated with a clonogenic cell survival assay. X-axis: dose of X-radiation (Gy). Y-axis: surviving fraction. The mean ± SEM is representative of two independent experiments carried out in duplicate. A *p* < 0.01 and *p* < 0.005 show significant differences

Since “cell death of cancer cells” was highlighted in *Vdac1*^*−/−*^ MEF (Additional file 2: Table S1A) in the IPA^TM^ analysis, we investigated whether the lack of *Vdac1* triggered apoptosis. While Wt MEF exhibited little apoptosis, basal apoptosis was persistent in normoxia (15 %) and hypoxia (20 %) in *Vdac1*^*−/−*^ MEF (Fig. 5a). The basal level of apoptosis was evaluated as 10 % of the nuclei of *Vdac1*^*−/−*^ MEF exhibiting blebbing in normoxia (Fig. 5b). Moreover, no cytochrome C release was observed for Wt MEF in normoxia, whereas *Vdac1*^*−/−*^ MEF showed release of cytochrome C in parallel to nuclear fragmentation (Additional file 13: Figure S8). The expression of Bak, Bax, and Mcl1 was similar in both cell lines and under both conditions (Fig. 5c). However, the expression of Bcl-X_L_ and Bcl-2, two anti-apoptotic members of the Bcl-2 family, was found to be reduced in *Vdac1*^*−/−*^ MEF, consistent with the basal apoptosis of these cells. In addition, Wt MEF incubated with the apoptotic stimulus staurosporine (STS) remained unaffected in both normoxia and hypoxia (Fig. 5d). However, *Vdac1*^*−/−*^ MEF were sensitive to STS in both conditions. We further confirmed that *Vdac1*^*−/−*^ MEF were sensitive to stimulated apoptosis and were also more sensitive to chemotherapy with doxorubicin and bleomycin than Wt MEF (Fig. 5e). No differences were observed for cisplatin. We also confirmed the protective effect of hypoxia in response to irradiation in Wt MEF (Fig. 5f) and found that *Vdac1*^*−/−*^ MEF in normoxia exhibited a level of radioresistance similar to that of Wt MEF in hypoxia. Moreover, *Vdac1*^*−/−*^ MEF showed more radioresistance in hypoxia than Wt MEF. Finally, as autophagy was also highlighted in *Vdac1*^*−/−*^ MEF by IPA^TM^ analysis (Additional file 3: Table S2A), we examined its induction in *Vdac1*^*−/−*^ MEF. We observed a higher background level of autophagy in *Vdac1*^*−/−*^ MEF compared to Wt MEF (Additional file 14: Figure S9A-B).

### *Vdac1*^*−/−*^ RAS^V12^-transformed MEF form inflammatory tumors that grow faster than Wt tumors

**Fig. 6:**
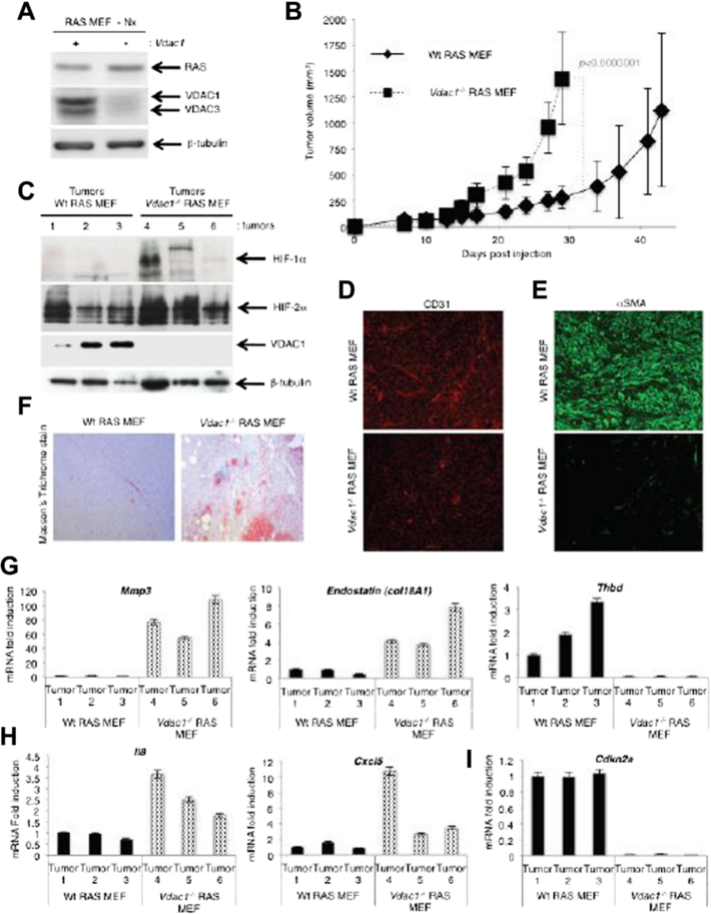
In vivo allograft tumor growth of RASV12-transformed MEF expressing (Wt RAS MEF) or not (*Vdac1* ^−/−^ RAS MEF) VDAC1. **a** Wt RAS (+) and *Vdac1* ^−/−^ RAS (−) MEF were incubated in Nx for 24 h and cell lysates were analyzed by immunoblotting for RAS and VDAC. β-tubulin was used as a control. **b** Allograft tumor growth of Wt RAS MEF and *Vdac1* ^−/−^ RAS MEF injected into one of the flanks of nod-scid mice. Five mice were studied per condition. A *p* < 0.0000001 shows significant differences. **c** Wt RAS (tumors 1, 2, and 3) and *Vdac1* ^−/−^ RAS MEF-derived tumors (tumors 4, 5, and 6) cell lysates were analyzed by immunoblotting for HIF-1α, HIF-2α, and VDAC. β-tubulin was used as a loading control. **d** Immunofluorescence of CD31 to detect blood vessels in Wt RAS and *Vdac1* ^−/−^ RAS MEF-derived tumors. **e** Immunofluorescence of α-smooth muscle actin to visualize the structure of blood vessels in Wt RAS and *Vdac1* ^−/−^ RAS MEF-derived tumors. **f** Representative images of Masson’s trichrome stained Wt RAS and *Vdac1* ^−/−^ RAS MEF-derived tumors. Red staining indicates red blood cells. **g** Expression of the mRNA of *Mmp3, Col18A1* (*Endostatin*) and *Thbd* in Wt RAS (Wt RAS MEF) and *Vdac1* ^−/−^ RAS MEF-derived tumors (*Vdac1* ^−/−^ RAS MEF). **h** Expression of the pro-inflammatory cytokine mRNA of *Il8* and *Cxcl5* in Wt RAS (Wt RAS MEF) and *Vdac1* ^−/−^ RAS MEF-derived tumors (*Vdac1* ^−/−^ RAS MEF). **i** Expression of the mRNA of *Cdkn2a* in Wt RAS (Wt RAS MEF) and *Vdac1* ^−/−^ RAS MEF-derived tumors (*Vdac1* ^−/−^ RAS MEF)

Wt and *Vdac1*^*−/−*^ RAS-transformed MEF showed similar expression of RAS (Fig. 6a), proliferation (Additional file 18: Figure S10A), type of morphology (Additional file 18: Figure S10B), rate of migration (Additional file 18: Figure S10C), level of glycolysis (Additional file 18: Figure S10D), and respiration (Additional file 18: Figure S10E) as non-transformed MEF. However, while the Wt RAS and *Vdac1*^*−/−*^ RAS MEF showed the same relative resistance to irradiation as previously observed (Fig. 5f), the basal level of resistance of transformed cells was much higher than that of non-transformed cells (Additional file 18: Figure S10F). Both Wt RAS or *Vdac1*^*−/−*^ RAS MEF plated in soft agar acquired the ability to grow under anchorage-independent conditions (Additional file 18: Figure S10G). Consistent with this, *Vdac1*^*−/−*^ RAS MEF more rapidly formed tumors in nod-scid mice than Wt RAS MEF (Fig. 6b). Most of the tumors derived from *Vdac1*^*−/−*^ RAS MEF reached a size of 1 cm^3^ within 27 days of injection, whereas Wt RAS MEF-derived tumors reached this size within 41–43 days and the tumor weights were similar (Additional file 18: Figure S10H). No metastases were detected at necropsy (data not shown). We noted that *Vdac1*^*−/−*^ RAS MEF-derived tumors showed bloody tumors with a soft texture while Wt RAS MEF-derived tumors had a harder texture (data not shown) (Additional file 15: Figure S11A). The level of HIF-1α and HIF-2α was higher in the three tumors derived from *Vdac1*^*−/−*^ RAS MEF than in Wt RAS MEF (Fig. 6c). Compared to Wt RAS MEF-derived tumors, there was a significance increase in the number of CD31-positive vessels (Fig. 6d) and number of vessels (Additional file 15: Figure S11B-C). Moreover, the immunofluorescence to alpha-smooth muscle actin was higher for Wt than for *Vdac1*^*−/−*^ RAS MEF tumors (Fig. 6e). Masson’s trichome staining showed a massive leak of red blood cells from blood vessels into the tumor tissue of the *Vdac1*^*−/−*^ RAS MEF (Fig. 6f) and more hemoglobine in the tumor (Additional file 15: Figure S11D), suggesting that the leak came from partial vascular remodeling, probably coupled with enhanced permeability. When comparing the non-transformed *Vdac1*^*−/−*^ to Wt MEF in the IPA^TM^ analysis for «Development of blood vessels», three genes were highly induced, matrix metalloproteinase-3 (*Mmp3*), platelet-derived growth factor β (*Pdgf*β), and collagen type XVIII alpha 1 (*Col18A1*), while one gene, thrombomodulin (*Thbd*), was highly repressed. Evaluation of the level of the mRNA confirmed the IPA^TM^ results with the exception of *Pdgf*β (Fig. 6g). From the IPA^TM^ analysis (Additional file 2: Table S1A-C, Additional file 3: Table S2A-C), the inflammatory response was central to loss of VDAC1. Moreover, as leakage may be associated with ROS production, generating an inflammatory response, we investigated the expression of pro-inflammatory genes. Increased expression of the pro-inflammatory cytokines interleukine 8 (*Il-8*) and chemokine (C-X-C motif) ligand 5 (*CxCl5*) were detected in *Vdac1*^*−/−*^ RAS MEF-derived tumors (Fig. 6h). Finally, we noted that cyclin-dependent kinase inhibitor 2A (*Cdkn2A*) (Fig. 1f, Additional file 6: Figure S1), a tumor suppressor gene, was markedly inhibited, likely via HIF-1α in normoxia and hypoxia in the absence of VDAC1. The expression level of *Cdkn2A* (Fig. 6i) was decreased likely favoring the rapid growth of *Vdac1*^*−/−*^ RAS MEF-derived tumors. Wt RAS MEF-derived tumors showed a much higher level of staining for VEGFA than *Vdac1*^−/−^ RAS MEF tumors, which correlated with the decrease in CD31-positive blood vessels in *Vdac1*^−/−^ RAS MEF tumors (Additional file 16: Figure S12). These results suggest a strong impact of VDAC1 on tumor development, probably through alterations in the inflammatory response as a result of an abnormal vasculature due to HIF-1α stabilization and ROS production.

## Discussion

Herein, we characterized the gene expression profile and cellular phenotype of *Vdac1*-deficient MEF in normoxia or hypoxia. Our results revealed important functions of VDAC1, in cultured cells and in in vivo allograft tumors, which were primarily driven by ROS and HIF-1.

The gene expression profiles of Wt vs *Vdac1*^*−/−*^ MEF in normoxia and hypoxia were compared keeping in mind that the other isoforms VDAC2 and VDAC3 may compensate [24]. However, we did not detect the VDAC2 protein, and the expression of VDAC3 protein remained constant. When comparing *Vdac1*^*−/−*^ to Wt MEF, the largest difference in expression was noted in normoxia, as HIF-1α and HIF-2α were already stabilized by ROS in *Vdac1*^*−/−*^ MEF. Therefore, the hypoxic effect on gene expression was diminished in *Vdac1*^*−/−*^ MEF. The mRNAs affected by the loss of VDAC1 are involved in cancer and metastasis, but also in fibrosis, neuromuscular disease and finally in the inflammatory response. The role of VDAC1 in cancer has only just started to be investigated [7, 8, 4]. The results of the present study reveal that the role of VDAC1 in cancer is connected to modulation of energy and cell death, which are closely interconnected. We noted a decrease in glycolysis in *Vdac1*^*−/−*^ MEF possibly due to the decrease in HKII expression. HKII has been shown to protect cancer cells from entering apoptosis by blocking the interaction of the pro-apoptotic protein Bax with VDAC1 [25]. Two critical anti-apoptotic factors, Bcl-X_L_ and Bcl-2, were expressed in *Vdac1*^*−/−*^ MEF. Bcl-X_L_ has been shown to critically regulate the opening of VDAC1 and to thus influence apoptosis, possibly through the permeability transition pore complex V [26-29]. Interestingly, in *Vdac1*^*−/−*^ MEF, neither Bcl-X_L_ nor Bcl-2, two critical anti-apoptotic factors, were expressed. As Bcl-X_L_ may influence metabolism [30], it is tempting to hypothesize that the absence of Bcl-X_L_, like HKII, may play a role in the reduced capacity to produce energy of *Vdac1*^*−/−*^ MEF in normoxia. A decrease in expression of HKII and Bcl-X_L_ would certainly affect apoptosis. Indeed, mitochondria-associated HKII has been shown to protect cancer cells from entering apoptosis by blocking the interaction of the pro-apoptotic protein Bax with VDAC1 [25]. Moreover, a decrease in the pool of anti-apoptotic proteins such as Bcl-X_L_ or Bcl-2 will inevitably destabilize the balance of anti-apoptotic/pro-apoptotic factors. We observed a basal level of apoptosis (around 10 % cell death), suggesting that VDAC1 is indispensable for mitochondrial cell death. This contrasts with data of a previous study using the same MEF [31].

In hypoxia in *Vdac1*^*−/−*^ MEF, we observed both enlarged mitochondria with abundant cristae and mitochondria similar to those in normoxia. The former may reflect the improved proliferation, thus better bioenergetics of the cells, and the later of mitochondria that had not or could not adapt. However, one question remains: how do these cells survive in normoxia with a low level of glycolysis and respiration? Fatty acid alpha oxidation by *Vdac1*^*−/−*^ MEF could be a possible explanation. The deficiency in *Vdac1* in both MEF and RAS-transformed MEF resulted in a reduction in proliferation in normoxia. Examination of the analytic technique for assessment of RNAi by similarity (ATARiS) [32], a computational method to assess the effects of gene suppression on cell survival/proliferation, in the Project Achilles database [33] supported this result. We specifically focused on cell lines with K-Ras mutations. The ATARiS gene-level score of dependency showed that knockout of *Vdac1* decreased proliferation, even in human cancer cells driven by oncogenic K-Ras (Additional file 17: Figure S13A). Based on these results, could *Vdac1* be a putative cancer driver gene in human cancer? The Cancer Genome Atlas (TCGA) data sets from 89 cancer studies were analyzed for gene mutation, deletion, gain, and amplification in the *Vdac1* gene with cBioPortal (http://www.cbioportal.org) [34, 35]. The alteration frequency varied from 80 to 1 % depending on the type of cancer. The *Vdac1* gene was mainly heterologously lost (light blue) and/or gained (pink). Homologous loss (dark blue) and amplification (red) were also observed, which suggested a putative role in development of human cancers (Additional file 17: Figure S13B). However, *Vdac1*^*−/−*^ MEF grew better than Wt MEF in hypoxia, and ERK activation was required. Chan et al. showed that ROS can participate in the degradation of DUSP6 [36]. Since ROS accumulated in *Vdac1*^*−/−*^ MEF, due in part to a decrease in GPX7 expression, DUSP6 was degraded in normoxia and hypoxia.

The data from *Vdac1*^*−/−*^ RAS MEF tumors in mice emphasized two major events: i) destructuration of blood vessels and ii) inflammation. The increase in endostatin, an inhibitor of angiogenesis that inhibits endothelial cell proliferation, migration/invasion, and tube formation, may explain the decrease in the number of blood vessels in these tumors. Moreover, the low level of VEGFA expression in the *Vdac1*^*−/−*^ RAS MEF tumors reinforced the implication of VDAC1 in vascular development. This newly identified role of VDAC1 in modulating the structure of blood vessels may explain why the expected Mendelian ratio of 1:2:1 was not observed for heterozygous mice (*Vdac1*^+/−^) [5]. Only 40 % of the expected number of *Vdac1*^−/−^ mice survived and, using timed matings, the fetal loss was determined to occur between embryonic day 10.5 and 11.5, a time during which blood vessel formation occurs. We speculate that 60 % of the *Vdac1*^−/−^ mice may develop dysfunctional blood vessels during embryogenesis. Why the other 40 % survive and show no obvious outward signs of deficiency remains obscure. The *Vdac1*^*−/−*^ RAS MEF tumors showed less blood vessels than Wt tumors and exhibited an inflammatory response that may result from infiltration of red blood cells (RBCs) through permeabilization of the remaining blood vessels. RBCs could serve as a potential source of ROS since they contain a large pool of O_2_ that is autoxidized in a hypoxic microenvironment, thereby promoting inflammation [37]. One of the early responses to tissue damage due to ROS is production of IL-8, a pro-inflammatory cytokine often associated with advanced stage cancer and with poor prognosis. RBCs can also bind inflammatory mediators such as IL-8. The mRNA level of *Il-8* and *Cxcl5* were markedly elevated in *Vdac1*^*−/−*^ RAS MEF, which supports the hypothesis of an enhanced inflammatory response. Finally, it has been shown that RBCs may induce the secretion of matrix metalloproteinases such as *mmp-3* by fibroblasts [38], which impacts on tissue remodeling. Thrombomodulin, a protein that maintains vascular homeostasis via its anti-inflammatory properties [39], was not expressed in the *Vdac1*^*−/−*^ RAS MEF, so the pro-inflammatory activity is likely not repressed. The cascade of events leading to tumor development was accompanied by a notable decrease in the mRNA level of *Cdkn2A*, a tumor suppressor gene.

While we observed more rapid tumor growth of *Vdac1*^*−/−*^ RAS MEF compared to Wt RAS MEF, a study using A549 cells (human lung adenocarcinoma epithelial cells) reported that the silencing of *Vdac1* expression with siRNA inhibited cancer cell proliferation and tumor growth in vivo [40]. However, we used a mouse/mouse system (tumor/host) while Arif et al. used a human/mouse system (tumor/host). In addition, we used MEF rather than lung cancer cells. Finally and most importantly, our system was a total knockout of *Vdac1* and not on partial and temporary silencing as for siRNA. Is angiogenesis induced in the same way in these tumors? We suggest that the *Vdac1*^*−/−*^ RAS MEF adapted and offset an imbalance in some metabolic, angiogenic, or inflammatory processes to proliferate.

## Conclusions

**Fig. 7:**
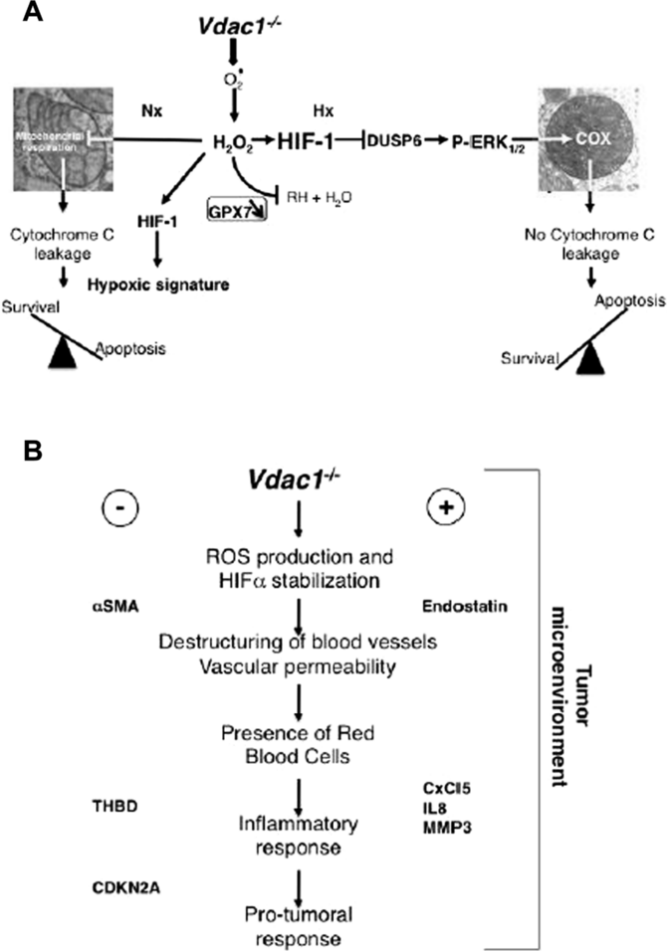
Schematic representation of the impact of *Vdac1* knockout (*Vdac1* ^−/−^) on proliferation and cell death of MEF in vitro (**a**) and tumor growth in vivo (**b**). **a** In normoxia (Nx), the knockout of *Vdac1* was associated with production of ROS that reduced proliferation and increased apoptosis (cytochrome C leakage). In hypoxia (Hx), the knockout of *Vdac1* maintained the production of ROS but the effect was offset by inhibition of DUSP6 while allowing slight activation of P-ERK. Activated P-ERK effected the activity of COX7A1 and COX4-2, while promoting proliferation. **b** Through production of ROS, which stabilizes HIF-1α, a cascade of events induced (+) or repressed (−) processes that destabilized blood vessels and induced an inflammatory response. This cascade will then lead to a pro-tumoral response. αSMA: alpha-smooth muscle actin; THBD: thrombomodulin; CDKN2A: cyclin-dependent kinase inhibitor 2A; CxCL5: chemokine (C-X-C motif) ligand 5; IL8: interlukin 8; MMP3: matrix metallopeptidase 3

Our study demonstrates that VDAC1 is not just a pore that allows passage of metabolites; it is a major mitochondrial protein that controls crucial processes involved in vital functions such as metabolism and cell death (Fig. 7). This study provides a rationale for investigating VDAC1 as a therapeutic target in both normoxic and hypoxic tissues with tumor characteristics.

## Additional files

Additional file 1:
**Supplemental Materials and Methods.**
Additional file 2: Table S1. Main biological functions associated with the alterations of *Vdac1* ^−/−^ MEF compared to Wt MEF (Wt) in normoxia (Nx) or hypoxia (Hx). Significant categories of (A) diseases and functions, (B) canonical pathways, and (C) upstream regulators associated with the comparison of wild-type (Wt) and *Vdac1* ^−/−^ MEF in Nx or Hx using IPA^TM^. Activation z-score (cut-off = 2) or –log10 *p* value (cut-off = 2) are represented. Additional file 3: Table S2. Main biological functions associated with the comparison of hypoxic and normoxic conditions in Wt MEF or *Vdac1* ^−/−^ MEF. Significant categories of (A) diseases and functions, (B) canonical pathways, and (C) upstream regulators associated with the comparison of Normoxia (Nx) and hypoxia (Hx) in wild-type (Wt) or *Vdac1* ^−/−^ MEF in using IPA^TM^. Activation z-score (cut-off = 2) or –log10 *p* value (cut-off = 2) are represented. Additional file 4: Table S3. Mus musculus dual specificity phosphatase (DUSP) mRNA levels associated with the comparison of hypoxic and normoxic conditions in Wt MEF or *Vdac1* ^−/−^ MEF. Additional file 5: Table S4. Mus musculus superoxide dismutase (Sod), glutathione peroxidase (GPX), and catalase (Cat) mRNA levels associated with the comparison of hypoxic and normoxic conditions in Wt MEF or *Vdac1* ^−/−^ MEF. Additional file 6: Figure S1. HIF-1α signaling pathway in hypoxic *Vdac1* ^−/−^ MEF vs Wt MEF. Red and green color codes for up- and down-regulation, respectively. Additional file 7: Figure S2. Perturbations in OXPHOS in *Vdac1* ^−/−^ MEF. Ingenuity pathway analysis of the OXPHOX in *Vdac1* ^−/−^ vs Wt MEF in Nx and Hx. Green color codes for down-regulation. Additional file 8: Figure S3. Cytochrome oxidase expression: (A) Immunofluorescence to COX4-2 in Wt and *Vdac1* ^−/−^ MEF in Nx for 72 h and (B) COX4-2 in Wt and *Vdac1* ^−/−^ MEF in Hx for 72 h in the absence (Ctl) or presence of the MEK inhibitor (PD184352). Additional file 9: Figure S4. Reactive oxygen species status of Wt and *Vdac1* ^−/−^ MEF. (A) Mitochondrial hydrogen peroxide production. (B) Immunoblotting for SOD1, SOD2, and SOD3 of Wt (+) and *Vdac1* ^−/−^ (−) MEF incubated in normoxia or hypoxia for 72 h. β-tubulin was used as a loading control. Additional file 10: Figure S5. Glutathione peroxidase expression and the effect of ebselen. (A) Immunofluorescence to GPX7 in Wt and *Vdac1* ^−/−^ MEF in Nx or Hx for 72 h. (B) Immunoblotting for HIF-1α of Wt and *Vdac1* ^−/−^ MEF in the absence or presence of ebselen in normoxia. (C) Proliferation of Wt and *Vdac1* ^−/−^ MEF in the absence or presence of ebselen in normoxia. (D) Viability of Wt and *Vdac1* ^−/−^ MEF in the absence or presence of ebselen in normoxia. Additional file 11: Figure S6. Changes in metabolic pathways in *Vdac1* ^−/−^ vs Wt MEF. Ingenuity pathway analysis of the metabolic activity of *Vdac1* ^−/−^ vs Wt MEF in (A) normoxia and (B) hypoxia. Red color code for up- regulation. Additional file 12: Figure S7. Glucose and glutamine metabolism. (A) Wt and *Vdac1* ^−/−^ MEF were cultured for 2 days in Nx or Hx in the presence of 2-deoxy-d-glucose (2DG, 1 μM) or without glucose (−Gluc). The cell number was measured on a Beckman Coulter apparatus (squares). The percentage of cell mortality was measured by trypan blue exclusion (black dots). The mean ± SEM is representative of two independent experiments carried out in duplicate. (B) The glutamate concentration in the media of Wt and *Vdac1* ^−/−^ MEF cultured in normoxia (Nx) or hypoxia 1 % O_2_ (Hx) was measured at day 7 using a YSI 2700 apparatus. *Vdac1* ^−/−^ MEF convert little or no glutamine into glutamate compared to Wt MEF. Additional file 13: Figure S8. Knockout of *Vdac1* significantly disrupted apoptosis. Immunofluorescence to cytochrome C and DAPI in Wt and *Vdac1* ^−/−^ MEF in Nx. Additional file 14: Figure S9. Autophagy was detected in *Vdac1* ^−/−^ MEF. (A) Representative images of electron micrographs of Wt and *Vdac1* ^−/−^ MEF incubated in normoxia (Nx) for 24 h. *Vdac1* ^−/−^ MEF showed autophagosomes with double membranes (arrow). (B) Wt and *Vdac1* ^−/−^ MEF lysates were analyzed in the absence (−) or presence of chloroquine (CQ) by immunoblotting to LC3. ARD1 was used as a loading control. The ratio of LC3-II/ARD1 was measured. Additional file 15: Figure S11. Red blood cells are present in *Vdac1* ^−/−^ RAS MEF tumor tissue. (A) 20 mg of tumor tissue derived from Wt (1, 2, and 3) and *Vdac1* ^−/−^ RAS MEF (4, 5, and 6) tumors were resuspended in RLT buffer before DNA/RNA/protein extraction. (B) Quantification of the number of blood vessels/microscopic field of CD31-positive vessels ± SEM of tumor tissue derived from Wt RAS (1, 2, and 3) and *Vdac1* ^−/−^ RAS MEF (4, 5, and 6). (C) Average of the quantification of CD31-positive vessels ± SEM per microscopic field in Wt RAS MEF-derived tumors (Wt RAS MEF) and *Vdac1* ^−/−^ RAS MEF-derived tumors (*Vdac1* ^−/−^ RAS MEF). Statistical significance, *p* < 0.0001. (D) Immunofluorescence to hemoglobin (Hgb) in *Vdac1* ^−/−^ RAS MEF. Additional file 16: Figure S12. Immunohistochemical staining for VEGFA of tumor sections. (A) Negative control using secondary antibody only on Wt RAS MEFs. (B) VEGFA immunodetection in sections of Wt and *Vdac1* ^−/−^ RAS MEF-derived tumor using 4X (top panels), 20X (middle panels), and 40X (bottom panels) magnification. Additional file 17: Figure S13. VDAC1 expression in human tissues and cancers. (A) Heatmaps run from blue (low values, more dependency/essentiality) to white to red (high values, less dependency/essentiality). ATARiS gene-level data are median centered and therefore relative within the particular gene that is being viewed. (B) The Cancer Genome Atlas (TCGA) data sets of 89 cancer studies analyzed for mutation, deletion, gain, and amplification in the *Vdac1* gene with cBioPortal. Additional file 18: Figure S10. Characterization of RASV12 transformed MEF expressing (WT RAS MEF) or not (*Vdac1* ^−/−^ RAS MEF) V*dac1*. (A) Characterization of the growth of Wt and *Vdac1* ^−/−^ RAS MEF incubated in Nx or Hx for the indicated number of days. The mean ± SEM is representative of four independent experiments carried out in duplicate. A *p* < 0.00001 shows significant difference from the normoxia for *Vdac1* ^−/−^ RAS MEF. (B) Representative phase contrast photographs of Wt and *Vdac1* ^−/−^ RAS MEF incubated in Nx for 72 h. Scale bars represent X μm. (C) Relative migration of Wt and *Vdac1* ^−/−^ RAS MEF in Nx as evaluated in a xCELLigence system. The mean ± SEM is representative of two independent experiments carried out in quadruplicate. (D) The extracellular acidification rate (ECAR) in Nx of Wt and *Vdac1* ^−/−^ RAS MEF was evaluated with a Seahorse XF bioenergetic system. Glucose (Glu 10 mM) and oligomycin (Oligo 1 μM) were injected at the indicated times. (E) The oxygen consumption rate (OCR) in Nx for Wt and *Vdac1* ^−/−^ RAS MEF was measured in real time with a Seahorse XF. Glucose (Glu 10 mM), oligomycin (Oligo 1 μM), carbonilcyanide p-triflouromethoxyphenylhydrazone (FCCP 1 μM), and Rotenone/Antimycine A (Rot/AA, 1 μM/1 μM) were injected at the indicated times. The mean ± SEM is representative of three independent experiments carried out in quadruplicate. (F) Radioresistance of Wt and *Vdac1* ^−/−^ RAS MEF cultured for 24 h in Nx or Hx and treated with the indicated dose of radiation. Cell growth was then evaluated with a clonogenic cell survival assay. X-axis: dose of X-radiation (Gy). Y-axis: surviving fraction. The mean ± SEM is representative of two independent experiments carried out in duplicate. (G) Soft agar assay of Wt and *Vdac1* ^−/−^ RAS MEF. (H) Tumor weight of Wt (Wt RAS MEF) and *Vdac1* ^−/−^ RAS MEF-derived tumors (*Vdac1* ^−/−^ RAS MEF).

## Abbreviations

2-Deoxy-d-glucose; αSMA: alpha-smooth muscle actin
ARD1: arrest defective 1
ATARiS: analytic technique for assessment of RNAi by similarity
CxCL5: chemokine (C-X-C motif) ligand 5
CDKN2A: cyclin-dependent kinase inhibitor 2A
COX: cytochrome oxidase
DUSP: dual specificity phosphatase
ECAR: extracellular acidification rate
ERK: extracellular signal-regulated kinase
GPX: glutathione peroxidase
Hx: hypoxia
HIF-1: hypoxia-inducible factor-1
HK: hexokinase
IPA^TM^: ingenuity pathway analysis^TM^
IL8: interlukin 8
NAC: N-acetyl-l-cysteine
Nx: normoxia
MMP3: matrix metallopeptidase 3
MEF: mouse embryonic fibroblasts
OCR: oxygen consumption rate
ROS: reactive oxygen species
SOD: superoxide dismutase
TCGA: The Cancer Genome Atlas
THBD: thrombomodulin
VDAC: voltage-dependent anion channel
