# Supplementary material for: Knockout of Vdac1 activates hypoxia-inducible factor through reactive oxygen species generation and induces tumor growth by promoting metabolic reprogramming and inflammation
Source: Cancer Metab. 2015 Aug 26;3:8. doi: 10.1186/s40170-015-0133-5 (PMC4551760; doi:10.1186/s40170-015-0133-5)
Supplement: Additional file 1: — Supplemental Materials and Methods. [file 40170_2015_133_MOESM1_ESM.pdf]

Supplemental materials and methods

Electron microscopy

Cells were fixed *in situ* with 1.6% glutaraldehyde in 0.1M phosphate buffer at room temperature (RT) and stored overnight at 4°C. Samples were rinsed in the same buffer and then postfixed with 1% osmium tetroxide and 1% potassium ferrocyanide in 0.1M cacodylate buffer for 1h at RT to enhance the staining of cytoplasmic membranes. Cells were rinsed with distilled water, embedded in epoxy resin, sectioned and examined with a Philips CM12 transmission electron microscope equipped with an Olympus SIS CCD camera. The area of the mitochondria of cells was calculated as an ellipse (0.785ab; n.2, 50 mitochondria per experiment; mean ± SD).

Colony-forming assay

Cells (5000–10000) were plated on 60mm dishes and incubated at 37°C, 5% CO_2 for colony formation. After 10 days, colonies were fixed with 10% (v/v) methanol for 15min and stained with 5% Giemsa (Sigma) for 30min for colony visualization.

ATP determination

MEFs (Wt and *Vdac1*$^{-/-}$) and MEF RAS (Wt and *Vdac1*$^{-/-}$) were incubated in hypoxia for 72h and then lysed. Quantification of ATP was done using a luciferin/luciferase-based assay (Cell Titer Glo kit, Promega) according to the manufacturer's instructions and results are expressed as relative luminescence units (RLU). Each condition was tested eight times and the entire experiment was done twice.

Lactate measurement

The lactate concentration in the supernatant of cells incubated either in normoxia or hypoxia for 48h was determined by an enzyme-based assay using 900µM β-NAD (BioChemika), 175µg/ml L-lactate dehydrogenase (BioChemika) and 100µg/ml glutamate-pyruvate transaminase (Roche) were diluted in a sodium carbonate (620mM)-L-gultamate (79mM) buffer adjusted to pH10. Lithium lactate was used as a standard. Measurement was done with a microplate reader after incubation for 30min at 37°C. For each condition, the protein concentration was determined to express the lactate concentration as mmole/µg protein.

Respirometry and extracellular acidification

The cellular oxygen consumption rate (OCR) and extracellular acidification rate (ECAR) were obtained using a Seahorse XF96 extracellular flux analyzer from Seahorse Bioscience (North Billerica, MA, USA). The final concentrations of the agents are given in the legends. Experiments

were performed according to the manufacturer's instructions. Protein standardization was performed after each experiment, with no noticeable differences in protein concentration and cell phenotype.

Determination of glutamate production

Glutamate concentrations, were measured in media supernatants using a Ysi 2300 STAT Plus analyzer (YSI Life Sciences). Measurements were done in triplicate and the experiment was repeated two times.

Caspase activation

Quantification of the caspase 3/7 activity was done using a luciferin/luciferase based assay (Caspase-Glo 3/7 kit, Promega) according to the manufacturer's instructions. Each condition was performed eight times and the entire experiment was done three times. Significant differences are based on the Student's *t* test * $p <0.005$. STS was added 4h prior to assay for caspase 3/7 activity,

Flow cytometry and ROS measurement

For measuring intra-cellular cytosolic ROS levels, cells were cultured in hypoxia or normoxia for 48h. Cells were then treated with oxidation sensitive DCF-DA (a final concentration of 10µM) in culture medium for 30 min at 37 °C. After trypsination, the fluorescence of DCF in cells was measured using a fluorescence-activated cell sorter (BD□FACSCALIBUR, analyzer).

Immunoblotting

Cells were lysed in 1.5x SDS buffer and the protein concentration determined using the BCA assay. 40µg of protein of whole cell extracts was resolved by SDS-PAGE and transferred onto a PVDF membrane (Millipore). Membranes were blocked in 5% non-fat milk in TN buffer (50mM Tris-HCl pH7.4, 150mM NaCl) and incubated in the presence of the primary and then secondary antibodies in 5% non-fat milk in TN buffer.

The Bak antibody was purchased from Abcam, Bid and tBid from (R&D Systems), Bax and Bcl-X_L from Santa Cruz, Bcl-2 from Novus and Mcl-1 from Sigma. Rat anti-mouse CD31 (MEC 13.3) was from BD Bioscience (San Diego, CA). VDAC1 antibody (ab15895) was purchased from Abcam. Rabbit polyclonal anti-HIF-1α antibody (antiserum 2087) was produced and characterized in our laboratory {Richard, 1999 #13}. ECL signals were normalized to either β-tubulin or ARD1 {Bilton, 2005 #388}. After washing in TN buffer containing 1% Triton-X100 and then in TN buffer, immunoreactive bands were visualized with the ECL system (Amersham Biosciences).
