# Supplementary material for: Knockout of Vdac1 activates hypoxia-inducible factor through reactive oxygen species generation and induces tumor growth by promoting metabolic reprogramming and inflammation
Source: Cancer Metab. 2015 Aug 26;3:8. doi: 10.1186/s40170-015-0133-5 (PMC4551760; doi:10.1186/s40170-015-0133-5)
Supplement: Additional file 2: Table S1. — Main biological functions associated with the alterations of Vdac1 −/− MEF compared to Wt MEF (Wt) in normoxia (Nx) or hypoxia (Hx). Significant categories of (A) diseases and functions, (B) canonical pathways, and (C) upstream regulators associated with the comparison of wild-type (Wt) and Vdac1 −/− MEF in Nx or Hx using IPATM. Activation z-score (cut-off = 2) or –log10 p value (cut-off = 2) are represented. [file 40170_2015_133_MOESM2_ESM.pdf]

A - Top Diseases & Functions

Diseases and Bio Functions	z-score Nx	z-score Hx
Accumulation of granulocytes	2.26	0.00
Activation of leukocytes	2.10	0.91
Activation of myeloid cells	2.26	1.06
Cancer	2.03	2.03
Cell death of cancer cells	0.00	2.38
Chemotaxis of antigen presenting cells	-0.37	-2.05
Digestive organ tumor	1.40	3.55
Efflux of lipid	0.00	-2.04
Epithelial neoplasia	1.14	2.16
Epithelial-mesenchymal transition of tumor cell lines	0.00	2.03
Fibrosis	2.00	0.49
Flux of lipid	0.00	-2.22
Neoplasia of carcinoma cell lines	0.00	2.43
Neuromuscular disease	-1.66	-2.21
Polarization of leukocytes	2.01	0.00
Quantity of bone marrow cells	0.00	-2.02
Recruitment of leukocytes	-0.68	-2.27
Response of antigen presenting cells	-2.13	0.00
Signaling of cells	-2.07	0.00
Signaling of tumor cell lines	-2.22	0.00
Transmigration of cells	0.00	-2.44

B - Top Canonical pathways

Canonical Pathway	p-val Nx	p-val Hx
Acute Phase Response Signaling	1.05	2.22
Agranulocyte Adhesion and Diapedesis	3.41	1.80
Atherosclerosis Signaling	3.54	1.35
Bladder Cancer Signaling	2.71	1.63
Dopamine Degradation	2.40	0.58
Ethanol Degradation IV	2.58	1.24
Fatty Acid alpha oxidation	3.02	0.82
Granulocyte Adhesion and Diapedesis	4.86	3.27
Guanine and Guanosine Salvage I	1.18	2.61
Guanosine Nucleotides Degradation III	0.47	2.66
HIF1 alpha Signaling	1.77	2.03
Histamine Degradation	3.15	0.87
Inhibition of Matrix Metalloproteases	2.88	2.04
Leukocyte Extravasation Signaling	2.31	0.82
LPS/IL-1 Mediated Inhibition of RXR Function	3.85	1.13
LXR/RXR Activation	2.44	2.11
Neuroprotective Role of THOP1 in Alzheimer's Disease	2.18	0.99
Oxidative Ethanol Degradation III	2.78	1.38
Purine Nucleotides Degradation II (Aerobic)	0.36	2.07

Putrescine Degradation III	2.78	0.73
Retinoate Biosynthesis II	1.01	2.15
Role of Macrophages, Fibroblasts and Endothelial Cells in Rheumatoid Arthritis	2.48	1.85
Role of Osteoblasts, Osteoclasts and Chondrocytes in Rheumatoid Arthritis	3.09	2.35
Tryptophan Degradation X	2.78	0.73
VDR/RXR Activation	2.38	4.01

C - Top Upstream regulators

Upstream regulators	z-score Nx	z-score Hx
AGT	-2.41	-0.99
BMP4	-1.31	-2.05
BMP6	-2.65	-1.71
CCL5	0.00	-2.43
CD40LG	2.10	0.00
CDKN2A	0.00	-2.25
CSF2	-0.02	3.41
HGF	-2.16	0.88
HIF1A	0.59	2.52
HOXA9	2.00	0.00
IFN Beta	-1.23	-2.39
IFNB1	-1.02	-2.24
IFNG	0.12	-2.40
IL17RA	2.00	0.00
IRF7	-0.41	-2.54
MEF2C	0.00	2.22
MEOX2	-2.41	-0.52
MLXIPL	0.00	-2.21
NFIC	0.00	2.00
NFkB (complex)	2.67	1.56
NFKBIA	-1.56	-2.18
PDGF BB	-1.80	-2.69
POU4F1	-2.14	0.00
RELA	2.06	1.08
SATB1	1.20	2.24
SOX2	-2.00	0.00
STAT1	-0.36	-2.11
TAF4	-0.92	-2.16
TCF3	0.00	-2.89
TGFB1	-2.50	-0.08
Tnf (family)	2.24	2.02
TNFSF12	2.15	0.78
WNT5A	-1.41	-2.65

Supplemental Table 1. Main biological functions associated with the alterations of *Vdac1*$^{-/-}$ MEF compared to Wt MEF (Wt) in normoxia (Nx) or hypoxia (Hx). (**A**, **B**, **C**, and **D**): Significant

categories of (**A**) Diseases and functions (**B**) Canonical pathways and (**C**) Upstream regulators associated with the comparison of wild-type (Wt) and *Vdac1*$^{-/-}$ MEF in Nx or Hx using IPA^{TM}. Activation z-score (cut-off=2) or –log10 p-value (cut-off=2) are represented.
