# Supplementary material for: Knockout of Vdac1 activates hypoxia-inducible factor through reactive oxygen species generation and induces tumor growth by promoting metabolic reprogramming and inflammation
Source: Cancer Metab. 2015 Aug 26;3:8. doi: 10.1186/s40170-015-0133-5 (PMC4551760; doi:10.1186/s40170-015-0133-5)
Supplement: Additional file 3: Table S2. — Main biological functions associated with the comparison of hypoxic and normoxic conditions in Wt MEF or Vdac1 −/− MEF. Significant categories of (A) diseases and functions, (B) canonical pathways, and (C) upstream regulators associated with the comparison of Normoxia (Nx) and hypoxia (Hx) in wild-type (Wt) or Vdac1 −/− MEF in using IPATM. Activation z-score (cut-off = 2) or –log10 p value (cut-off = 2) are represented. [file 40170_2015_133_MOESM3_ESM.pdf]

A - Top Diseases & Functions

Diseases and Bio Functions	z-score Wt	z-score *Vdac1*$^{-/-}$
Activation of myeloid cells	0.00	-2.70
Alignment of chromosomes	-2.33	-2.55
Apoptosis of squamous cell carcinoma cell lines	2.77	0.00
Association of chromatin	-2.79	-2.61
Association of chromosome components	-2.36	-1.98
Autophagy of cells	0.00	2.33
Cell death of fibroblast cell lines	2.29	1.88
Cell death of kidney cells	2.61	1.94
Cell death of melanoma cell lines	0.00	-2.00
Cell movement	-3.26	0.00
Cell movement of phagocytes	0.00	-3.41
Cell movement of tumor cell lines	-3.41	0.00
Cell survival	-2.48	-0.85
Cell transformation	-2.71	0.00
Cell viability	-2.32	-0.44
Cell viability of myeloma cell lines	-3.05	0.00
Cell viability of tumor cell lines	-2.46	-0.61
Chemotaxis of myeloid cells	0.00	-2.87
Chemotaxis of phagocytes	0.00	-2.89
Chromosomal congression of chromosomes	-2.43	-2.22
Colony formation	-3.13	-1.89
Colony formation of cells	-3.06	-2.18
Colony formation of fibroblasts	-2.21	0.00
Colony formation of lung cancer cell lines	0.00	-2.74
Colony formation of tumor cell lines	-2.25	-1.08
Cycling of centrosome	-2.73	0.00
Cytosis	-1.58	-2.01
Death of embryo	3.19	0.00
Differentiation of osteoblasts	-2.20	0.00
Double-stranded DNA break repair of cells	-2.06	0.00
Entry into interphase	-2.47	-2.19
Entry into interphase of oocytes	-2.00	-2.00
Entry into interphase of tumor cell lines	-2.35	0.00
Entry into S phase	-2.02	-1.47
Entry into S phase of tumor cell lines	-2.81	0.00
Fibrogenesis	0.00	-2.03
Fusion of atlas	2.24	0.00
Hematological neoplasia	2.04	0.79
Inflammatory response	0.00	-2.65
Interphase	-1.29	-2.04
Invasion of breast cancer cell lines	-2.14	0.00
Invasion of cells	-3.26	-2.74
Invasion of tumor cell lines	-3.87	-2.55
Liver cancer	-2.25	-0.01
Liver tumor	-2.29	-0.35
Lymphohematopoietic cancer	2.24	1.11

Lymphoid cancer	2.17	1.30
Lymphomagenesis	2.17	1.30
M phase of tumor cell lines	-2.09	-2.09
Mammary tumor	-2.59	-0.92
Metabolism of DNA	-2.27	-1.51
Metastasis	-1.61	-2.01
Microtubule dynamics	-2.95	0.00
Migration of cells	0.00	-3.61
Migration of tumor cell lines	0.00	-2.03
Mitosis	-2.26	-0.81
Necrosis of kidney	2.15	1.60
Necrosis of muscle	-0.24	-2.03
Oligodactyly	2.62	0.00
Organismal death	0.00	2.10
Organization of cytoplasm	-2.82	0.00
Organization of cytoskeleton	-2.79	0.00
Orientation of chromosomes	-2.33	0.00
Premature senescence of fibroblasts	2.24	0.00
Proliferation of breast cancer cell lines	-2.58	0.00
Proliferation of cells	-5.14	0.00
Proliferation of connective tissue cells	-3.33	0.00
Proliferation of fibroblasts	-3.17	0.00
Proliferation of lung cancer cell lines	-3.88	0.00
Proliferation of pancreatic cancer cell lines	-2.88	0.00
Proliferation of tumor cell lines	-4.78	0.00
Repair of cells	-3.05	0.00
S phase of bone cancer cell lines	-2.20	0.00
S phase of fibroblast cell lines	0.00	-2.18
S phase of tumor cell lines	-2.65	-2.68
Senescence of fibroblasts	2.22	0.00
Size of embryo	-5.23	0.00
Transformation of fibroblast cell lines	-2.90	-0.40

B - Top Canonical pathways

Canonical Pathway	p-val Wt	p-val *Vdac1*$^{-/-}$
5-aminoimidazole Ribonucleotide Biosynthesis	0.70	2.89
Acute Phase Response Signaling	0.95	3.02
ATM Signaling	3.73	2.52
Cell Cycle Control of Chromosomal Replication	6.16	2.31
Cell Cycle: G2/M DNA Damage Checkpoint Regulation	5.35	2.89
Cyclins and Cell Cycle Regulation	3.16	1.91
DNA Double-Strand Break Repair by Homologous Recombination	3.61	1.24
Estrogen-mediated S-phase Entry	3.75	2.55
Folate Polyglutamylation	1.35	3.20
Folate Transformations I	0.88	2.89
GADD45 Signaling	2.13	2.72
GDP-L-fucose Biosynthesis II (from L-fucose)	2.29	0.00

Gluconeogenesis I	0.65	2.05
Glutathione Biosynthesis	0.00	2.89
Glutathione-mediated Detoxification	0.00	2.68
Glycolysis I	1.75	2.16
Hereditary Breast Cancer Signaling	4.01	1.56
HIF1alpha Signaling	0.00	2.41
IL-6 Signaling	0.73	2.02
Mismatch Repair in Eukaryotes	4.21	1.63
Mitotic Roles of Polo-Like Kinase	6.83	1.53
NRF2-mediated Oxidative Stress Response	0.78	2.83
p38 MAPK Signaling	0.78	2.48
p53 Signaling	0.75	4.84
Purine Nucleotides De Novo Biosynthesis II	0.25	3.33
RhoA Signaling	0.00	2.29
Role of BRCA1 in DNA Damage Response	4.81	2.37
Role of CHK Proteins in Cell Cycle Checkpoint Control	3.53	0.94
Role of Tissue Factor in Cancer	0.00	3.06
Salvage Pathways of Pyrimidine Deoxyribonucleotides	2.20	1.70
Superpathway of Cholesterol Biosynthesis	0.91	2.81
Tetrahydrofolate Salvage from 5,10-methenyltetrahydrofolate	0.51	3.20
TR/RXR Activation	2.71	0.76
tRNA Splicing	0.00	2.06

C - Top Upstream regulators

Upstream regulators	z-score Wt	z-score *Vdac1*$^{-/-}$
Ap1	0.00	2.10
ATF3	2.45	1.50
ATF4	2.37	1.69
CCND1	-1.79	-2.09
CDKN2A	4.95	3.43
CSF2	-6.82	-5.18
CTNN beta-TCF/LEF	0.00	-2.00
Cyclin E	-1.98	-2.21
DDIT3	1.47	2.22
DTX1	0.00	2.24
E2f	-3.53	-2.54
E2F1	-3.53	-1.87
E2F2	-3.41	-3.20
E2F3	-2.96	-3.12
EBF1	0.00	-2.38
EIF4G2	0.00	2.24
Endothelin	0.00	2.00
EPAS1	4.00	3.58
ESRRG	2.17	1.60
F2RL1	0.00	2.18
FLI1	-0.10	-2.04
FOXL2	2.22	-0.20
FOXM1	-4.51	-3.52

FOXO1	-2.05	-0.54
FOXO3	2.51	2.21
FOXO4	0.95	2.22
FZD9	-1.98	-2.41
GRP	-2.00	0.22
Hdac	-2.40	-3.14
HGF	-4.47	-2.96
HIF1A	3.13	4.33
HNF1A	3.05	0.00
HNF4A	2.03	0.23
HOXB3	0.00	-2.00
HTT	-3.24	-1.54
Ifn	-2.36	-2.74
IFN alpha/beta	-3.28	-4.33
IFN Beta	-2.12	-3.77
IFN type 1	-2.91	-3.64
IFNA2	-2.12	-3.83
IFNAR1	-1.69	-2.48
IFNAR2	-2.24	-2.65
IFNB1	-0.81	-2.90
IFNG	-0.55	-2.29
IFNK	0.00	-2.00
IFNL1	-1.60	-3.16
IGF2BP1	-2.45	-1.63
IL10	0.00	2.27
IL13	0.00	-2.75
INHA	3.10	2.18
Interferon alpha	-2.28	-3.92
IRF1	-0.66	-2.57
IRF3	-2.90	-4.45
IRF5	-2.76	-3.34
IRF7	-4.23	-5.62
JAG2	0.00	2.00
JUN	-1.83	-2.45
JUNB	1.12	2.07
JUND	-1.99	-2.60
KDM5B	3.57	3.79
LEF1	-2.37	-1.84
MAX	-2.80	-2.97
MED1	-2.36	-1.87
MEF2C	1.89	2.36
MNT	0.00	-2.24
MYB	-2.38	-2.56
MYBL2	-2.44	-2.64
MYC	-4.00	-3.68
NFKB1	2.53	0.52
Notch	2.42	0.96
NR1H3	-2.07	-0.42
NR3C2	-2.44	-0.26
NRG1	0.00	2.26

NUPR1	7.60	6.92
PRL	-2.98	-2.83
Rb	3.42	2.02
RB1	4.24	3.15
RBL1	3.62	2.02
SIN3A	0.00	-2.24
SMAD2	1.38	2.64
SMARCB1	2.29	1.16
SPDEF	2.83	1.57
SPP1	-2.10	-2.18
SREBF2	0.00	-2.31
STAT1	-2.03	-4.74
STAT2	-1.46	-2.81
STAT4	3.30	5.70
TCF3	3.30	2.96
TFEB	0.00	2.43
TGFB2	-0.14	2.14
TLR4	-0.99	-2.65
TLX1	-2.00	0.00
TNFSF13	2.45	2.65
TP53	4.22	1.23
TRIM24	4.49	6.38
Vegf	-4.31	-3.15

Supplemental Table 2. Main biological functions associated with the comparison of hypoxic and normoxic conditions in Wt MEF or *Vdac1*$^{-/-}$ MEF. (**A**, **B**, **C**, and **D**): Significant categories of (**A**) Diseases and functions (**B**) Canonical pathways and (**C**) Upstream regulators associated with the comparison of Normoxia (Nx) and hypoxia (Hx) in wild-type (Wt) or *Vdac1*$^{-/-}$ MEF in using IPA^{TM}. Activation z-score (cut-off=2) or –log10 p-value (cut-off=2) are represented.
