# Supplementary material for: Knockout of Vdac1 activates hypoxia-inducible factor through reactive oxygen species generation and induces tumor growth by promoting metabolic reprogramming and inflammation
Source: Cancer Metab. 2015 Aug 26;3:8. doi: 10.1186/s40170-015-0133-5 (PMC4551760; doi:10.1186/s40170-015-0133-5)
Supplement: Additional file 4: Table S3. — Mus musculus dual specificity phosphatase (DUSP) mRNA levels associated with the comparison of hypoxic and normoxic conditions in Wt MEF or Vdac1 −/− MEF. [file 40170_2015_133_MOESM4_ESM.pdf]

		Wt Hx/Nx	*Vdac1*$^{-/-}$ Hx/Nx	Nx *Vdac1*$^{-/-}$ /Wt	Hx *Vdac1*$^{-/-}$ /Wt
Dusp1	NM_013642	1.65	1.92	0.14	0.41
Dusp2	NM_010090	-0.22	-0.57	0.43	0.08
Dusp3	NM_028207	0.58	0.66	-0.01	0.08
Dusp4	NM_176933	0.01	1.99	-1.44	0.53
Dusp5	NM_001085390	-0.39	0.00	-0.71	-0.32
**Dusp6**	**NM_026268**	**-0.31**	**-1.26**	**-1.94**	**-2.89**
Dusp7	NM_153459	-0.83	-0.84	0.29	0.29
Dusp8	NM_008748	-0.52	0.05	0.80	1.37
Dusp9	NM_029352	-0.84	-1.48	-0.73	-1.37
Dusp10	NM_022019	0.65	1.25	-0.14	0.45
Dusp11	NM_028099	-0.77	-0.46	0.32	0.62
Dusp12	NM_023173	0.43	-0.09	1.88	1.36
Dusp13	NM_001007268	0.74	2.46	0.68	2.41
Dusp14	NM_019819	-1.37	0.60	1.26	3.24
Dusp15	NM_145744	0.85	-0.15	-0.08	-1.08
Dusp16	NM_130447	0.65	0.60	-0.02	-0.07
Dusp18	NM_173745	-0.84	-1.01	0.61	0.43
Dusp19	NM_024438	0.55	-0.37	-0.26	-1.18
Dusp21	NM_028568	-0.18	0.32	-0.23	0.28
Dusp22	NM_134068	0.90	-0.83	0.78	-0.95
Dusp23	NM_026725	0.33	-0.20	-1.86	-2.39
Dusp26	NM_025869	0.14	-0.42	0.81	0.25
Dusp28	NM_175118	0.17	-0.48	0.90	0.25

Supplemental Table 3. Mus musculus dual specificity phosphatase (DUSP) mRNA levels associated with the comparison of hypoxic and normoxic conditions in Wt MEF or *Vdac1*$^{-/-}$ MEF.
