# Supplementary material for: Knockout of Vdac1 activates hypoxia-inducible factor through reactive oxygen species generation and induces tumor growth by promoting metabolic reprogramming and inflammation
Source: Cancer Metab. 2015 Aug 26;3:8. doi: 10.1186/s40170-015-0133-5 (PMC4551760; doi:10.1186/s40170-015-0133-5)
Supplement: Additional file 5: Table S4. — Mus musculus superoxide dismutase (Sod), glutathione peroxidase (GPX), and catalase (Cat) mRNA levels associated with the comparison of hypoxic and normoxic conditions in Wt MEF or Vdac1 −/− MEF. [file 40170_2015_133_MOESM5_ESM.pdf]

		Wt Hx/Nx	*Vdac1*$^{-/-}$ Hx/Nx	Nx *Vdac1*$^{-/-}$ /Wt	Hx *Vdac1*$^{-/-}$ /Wt
Sod1	NM_011434	-0.07	-0.24	-0.23	-0.40
Sod2	NM_013671	-0.72	-1.00	-0.59	-0.87
Sod3	NM_011435	-1.68	-2.87	2.64	1.45
Gpx1	NM_008160	-0.12	-0.37	-0.64	-0.89
Gpx2	NM_030677	-0.10	0.36	-0.62	-0.17
Gpx3	NM_008161	-0.25	-0.15	-1.21	-1.11
Gpx4	NM_001037741	-0.20	0.15	0.40	0.75
Gpx5	NM_010343	-0.16	-0.09	-0.09	-0.02
Gpx6	NM_145451	0.20	0.19	0.00	-0.01
**Gpx7**	**NM_024198**	**-0.77**	**0.14**	**-5.88**	**-4.98**
Gpx8	NM_027127	0.19	-0.46	0.72	0.07
Cat	NM_009804	1.23	0.62	-0.41	-1.02

Supplemental Table 4. Mus musculus superoxide dismutase (Sod), glutathione peroxidase (GPX) and catalase (Cat) mRNA levels associated with the comparison of hypoxic and normoxic conditions in Wt MEF or *Vdac1*$^{-/-}$ MEF.
