# Supplementary material for: Knockout of Vdac1 activates hypoxia-inducible factor through reactive oxygen species generation and induces tumor growth by promoting metabolic reprogramming and inflammation
Source: Cancer Metab. 2015 Aug 26;3:8. doi: 10.1186/s40170-015-0133-5 (PMC4551760; doi:10.1186/s40170-015-0133-5)
Supplement: Additional file 6: Figure S1. — HIF-1α signaling pathway in hypoxic Vdac1 −/− MEF vs Wt MEF. Red and green color codes for up- and down-regulation, respectively. [file 40170_2015_133_MOESM6_ESM.pdf]

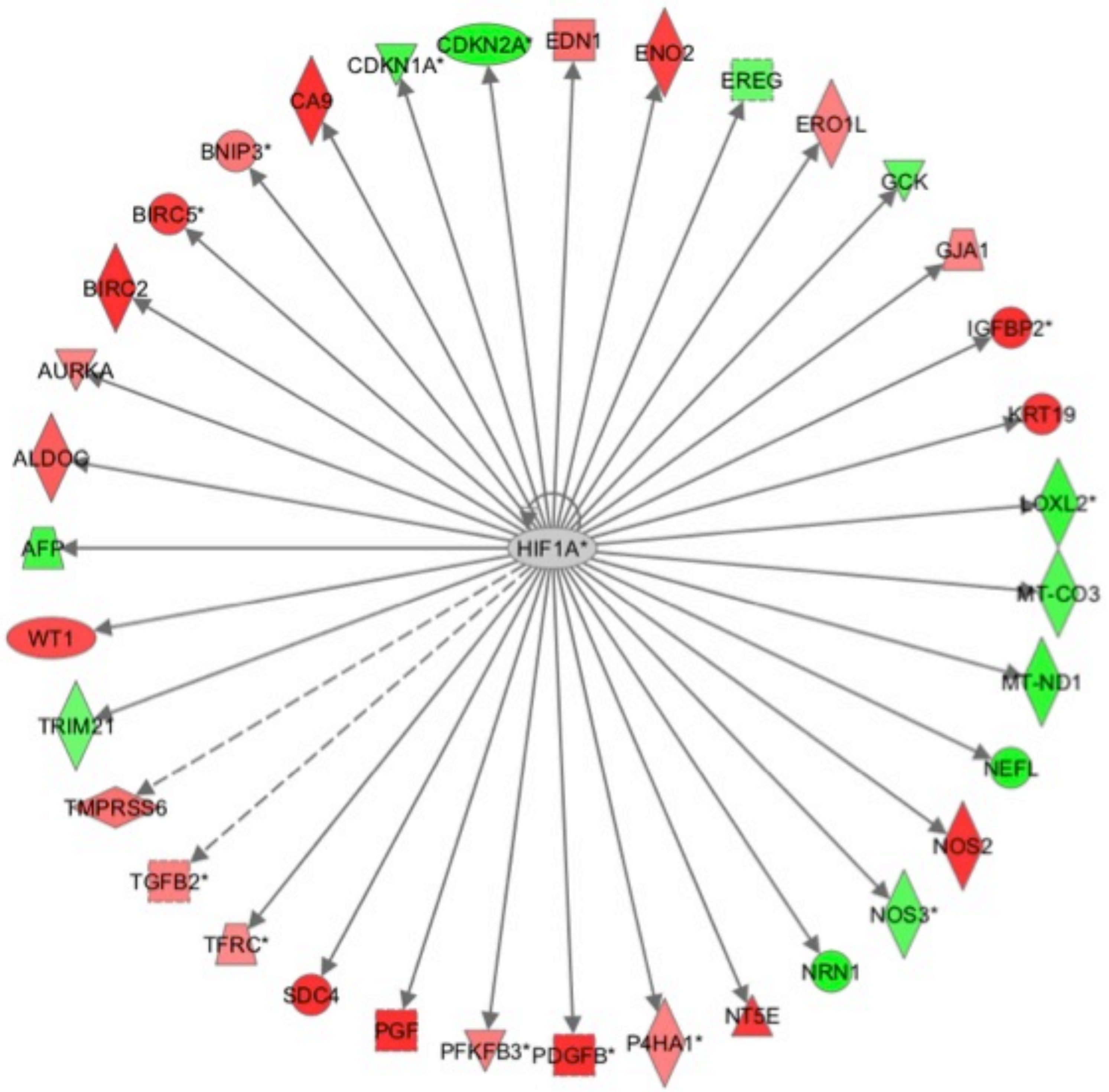

Supplemental Figure S1. HIF-1α signaling pathway in hypoxic *Vdac1*$^{-/-}$ MEF vs Wt MEF. Red and green color codes for up- and down-regulation, respectively.
