# Supplementary material for: Knockout of Vdac1 activates hypoxia-inducible factor through reactive oxygen species generation and induces tumor growth by promoting metabolic reprogramming and inflammation
Source: Cancer Metab. 2015 Aug 26;3:8. doi: 10.1186/s40170-015-0133-5 (PMC4551760; doi:10.1186/s40170-015-0133-5)
Supplement: Additional file 7: Figure S2. — Perturbations in OXPHOS in Vdac1 −/− MEF. Ingenuity pathway analysis of the OXPHOX in Vdac1 −/− vs Wt MEF in Nx and Hx. Green color codes for down-regulation. [file 40170_2015_133_MOESM7_ESM.pdf]

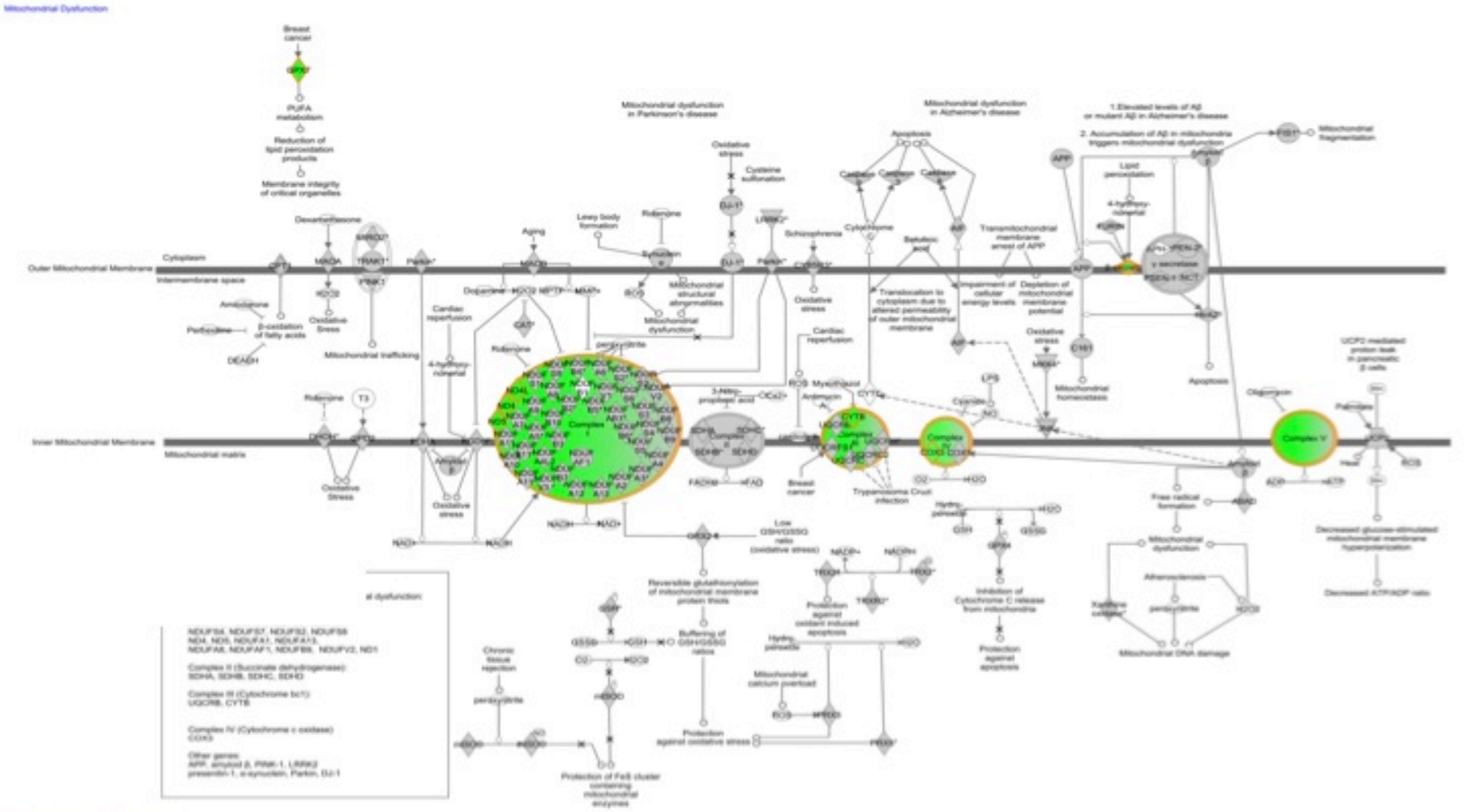

Supplemental Figure S2. Perturbations in OXPHOS in *Vdac1*$^{-/-}$ MEF. Ingenuity Pathway Analysis of the OXPHOX in *Vdac1*$^{-/-}$ *vs* Wt MEF in Nx and Hx. Green color codes for down-regulation.
