# Supplementary material for: Knockout of Vdac1 activates hypoxia-inducible factor through reactive oxygen species generation and induces tumor growth by promoting metabolic reprogramming and inflammation
Source: Cancer Metab. 2015 Aug 26;3:8. doi: 10.1186/s40170-015-0133-5 (PMC4551760; doi:10.1186/s40170-015-0133-5)
Supplement: Additional file 8: Figure S3. — Cytochrome oxidase expression: (A) Immunofluorescence to COX4-2 in Wt and Vdac1 −/− MEF in Nx for 72 h and (B) COX4-2 in Wt and Vdac1 −/− MEF in Hx for 72 h in the absence (Ctl) or presence of the MEK inhibitor (PD184352). [file 40170_2015_133_MOESM8_ESM.pdf]

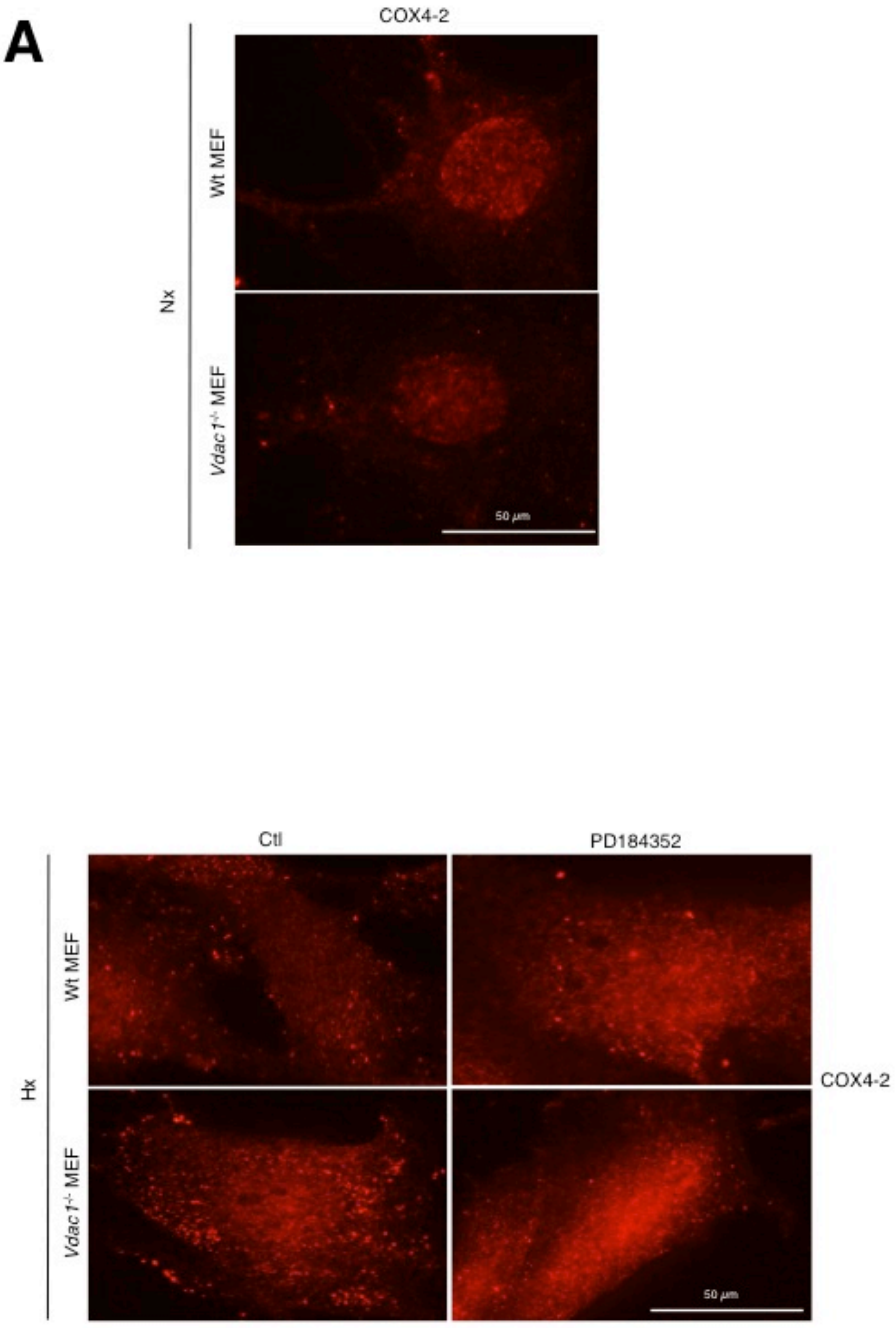

Supplemental Figure S3. Cytochrome oxidase expression: (A) Immunofluorescence to COX4-2 in Wt and *Vdac1*$^{-/-}$ MEF in Nx for 72h and **(B)** COX4-2 in Wt and *Vdac1*$^{-/-}$ MEF in Hx for 72h in the absence (Ctl) or presence of the MEK inhibitor (PD184352) .
