# Supplementary material for: Knockout of Vdac1 activates hypoxia-inducible factor through reactive oxygen species generation and induces tumor growth by promoting metabolic reprogramming and inflammation
Source: Cancer Metab. 2015 Aug 26;3:8. doi: 10.1186/s40170-015-0133-5 (PMC4551760; doi:10.1186/s40170-015-0133-5)
Supplement: Additional file 9: Figure S4. — Reactive oxygen species status of Wt and Vdac1 −/− MEF. (A) Mitochondrial hydrogen peroxide production. (B) Immunoblotting for SOD1, SOD2, and SOD3 of Wt (+) and Vdac1 −/− (−) MEF incubated in normoxia or hypoxia for 72 h. β-tubulin was used as a loading control. [file 40170_2015_133_MOESM9_ESM.pdf]

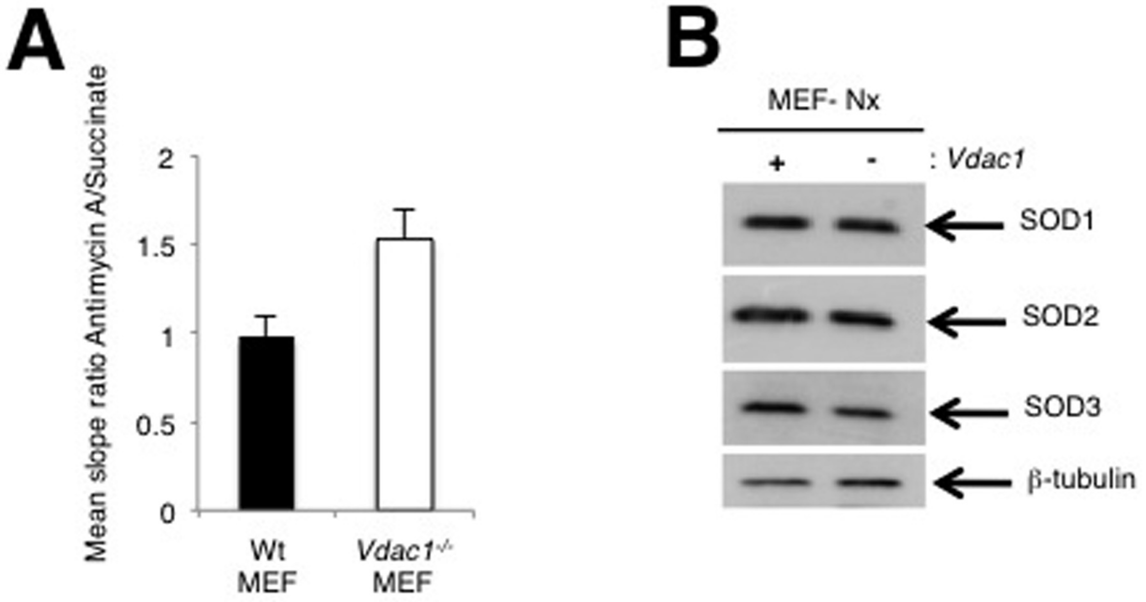

Supplemental Figure S4. Reactive oxygen species status of Wt and *Vdac1*$^{-/-}$ MEF. (A) Mitochondrial hydrogen peroxide production. **(B)** Immunoblotting for SOD1, SOD2 and SOD3 of Wt (+) and *Vdac1*$^{-/-}$ (-) MEF incubated in normoxia or hypoxia for 72h. β-tubulin was used as a loading control.
