# Supplementary material for: Knockout of Vdac1 activates hypoxia-inducible factor through reactive oxygen species generation and induces tumor growth by promoting metabolic reprogramming and inflammation
Source: Cancer Metab. 2015 Aug 26;3:8. doi: 10.1186/s40170-015-0133-5 (PMC4551760; doi:10.1186/s40170-015-0133-5)
Supplement: Additional file 10: Figure S5. — Glutathione peroxidase expression and the effect of ebselen. (A) Immunofluorescence to GPX7 in Wt and Vdac1 −/− MEF in Nx or Hx for 72 h. (B) Immunoblotting for HIF-1α of Wt and Vdac1 −/− MEF in the absence or presence of ebselen in normoxia. (C) Proliferation of Wt and Vdac1 −/− MEF in the absence or presence of ebselen in normoxia. (D) Viability of Wt and Vdac1 −/− MEF in the absence or presence of ebselen in normoxia. [file 40170_2015_133_MOESM10_ESM.pdf]

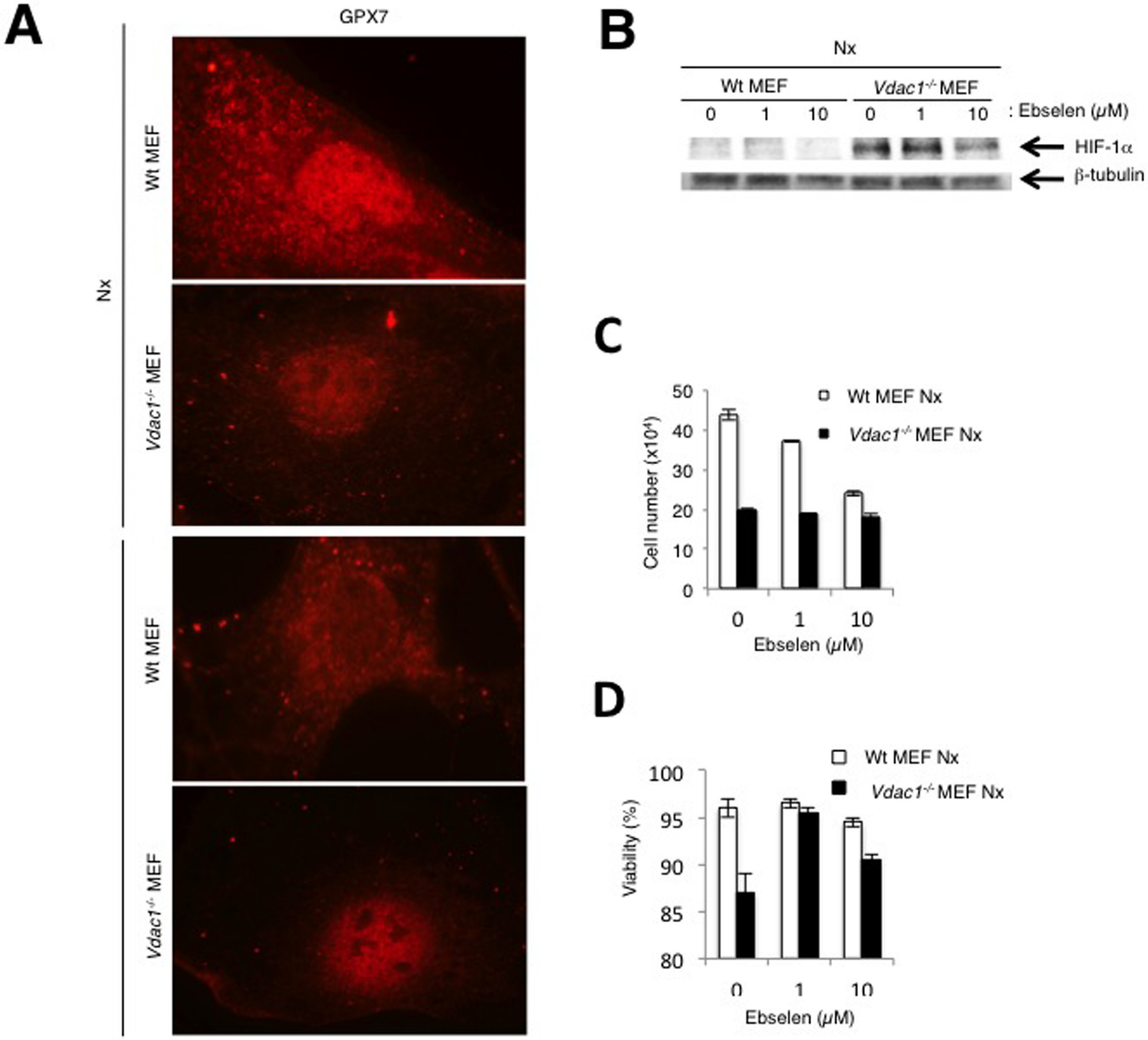

Supplemental Figure S5. Glutathione peroxidase expression and the effect of ebselen. (A) Immunofluorescence to GPX7 in Wt and *Vdac1*$^{-/-}$ MEF in Nx or Hx for 72h, **(B)** Immunoblotting for HIF-1α of Wt and *Vdac1*$^{-/-}$ MEF in the absence or presence of ebselen in normoxia. **(C)** Proliferation of Wt and *Vdac1*$^{-/-}$ MEF in the absence or presence of ebselen in normoxia. **(D)** Viability of Wt and *Vdac1*$^{-/-}$ MEF in the absence or presence of ebselen in normoxia.
