# Supplementary material for: Knockout of Vdac1 activates hypoxia-inducible factor through reactive oxygen species generation and induces tumor growth by promoting metabolic reprogramming and inflammation
Source: Cancer Metab. 2015 Aug 26;3:8. doi: 10.1186/s40170-015-0133-5 (PMC4551760; doi:10.1186/s40170-015-0133-5)
Supplement: Additional file 11: Figure S6. — Changes in metabolic pathways in Vdac1 −/− vs Wt MEF. Ingenuity pathway analysis of the metabolic activity of Vdac1 −/− vs Wt MEF in (A) normoxia and (B) hypoxia. Red color code for up- regulation. [file 40170_2015_133_MOESM11_ESM.pdf]

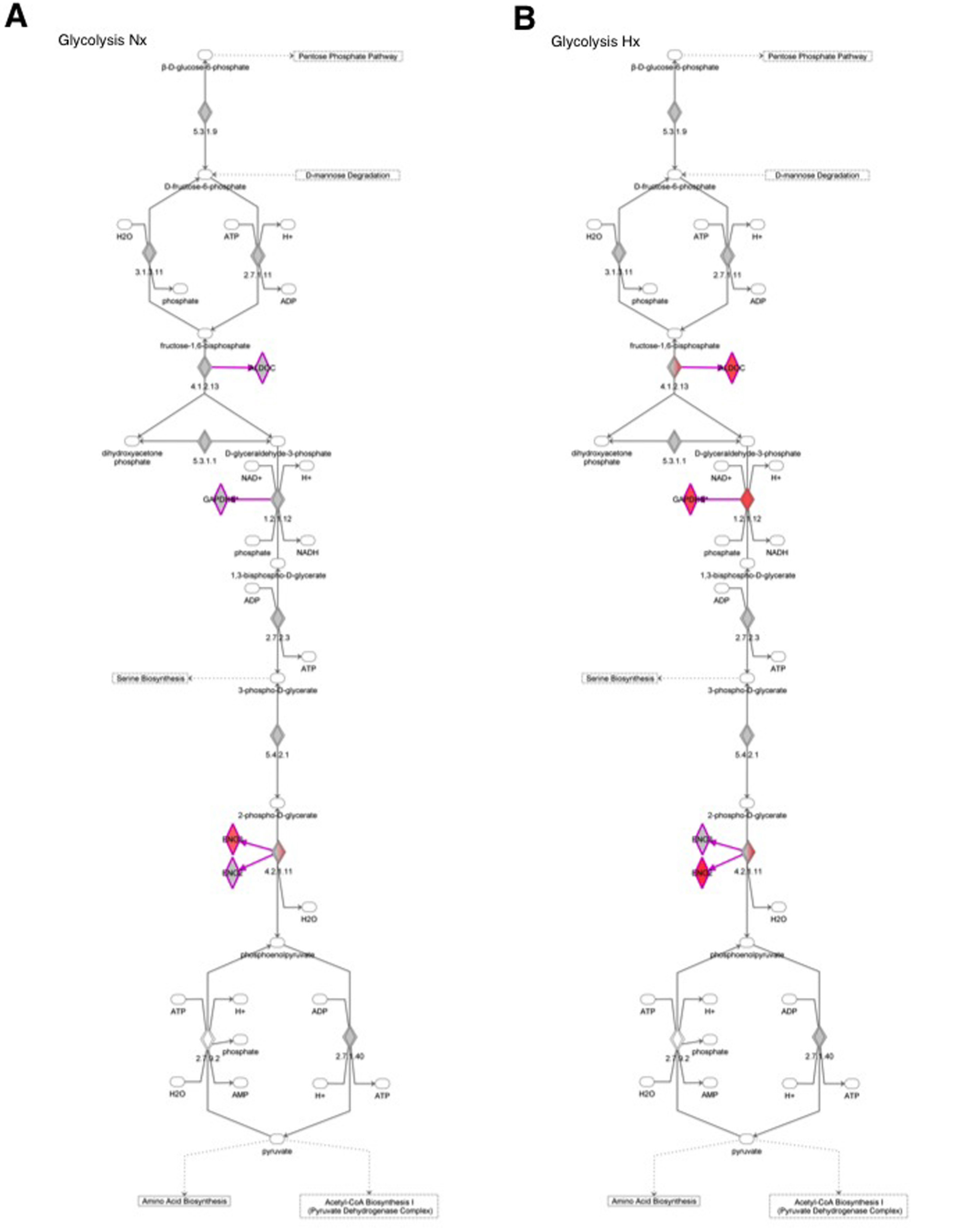

Supplemental Figure S6. Changes in metabolic pathways in *Vdac1*$^{-/-}$ vs Wt MEF. (A and B) Ingenuity Pathway Analysis of the metabolic activity of *Vdac1*$^{-/-}$ *vs* Wt MEF in (A) normoxia and (B) hypoxia. Red color code for up-regulation.
