# Supplementary material for: Knockout of Vdac1 activates hypoxia-inducible factor through reactive oxygen species generation and induces tumor growth by promoting metabolic reprogramming and inflammation
Source: Cancer Metab. 2015 Aug 26;3:8. doi: 10.1186/s40170-015-0133-5 (PMC4551760; doi:10.1186/s40170-015-0133-5)
Supplement: Additional file 12: Figure S7. — Glucose and glutamine metabolism. (A) Wt and Vdac1 −/− MEF were cultured for 2 days in Nx or Hx in the presence of 2-deoxy-d-glucose (2DG, 1 μM) or without glucose (−Gluc). The cell number was measured on a Beckman Coulter apparatus (squares). The percentage of cell mortality was measured by trypan blue exclusion (black dots). The mean ± SEM is representative of two independent experiments carried out in duplicate. (B) The glutamate concentration in the media of Wt and Vdac1 −/− MEF cultured in normoxia (Nx) or hypoxia 1 % O2 (Hx) was measured at day 7 using a YSI 2700 apparatus. Vdac1 −/− MEF convert little or no glutamine into glutamate compared to Wt MEF. [file 40170_2015_133_MOESM12_ESM.pdf]

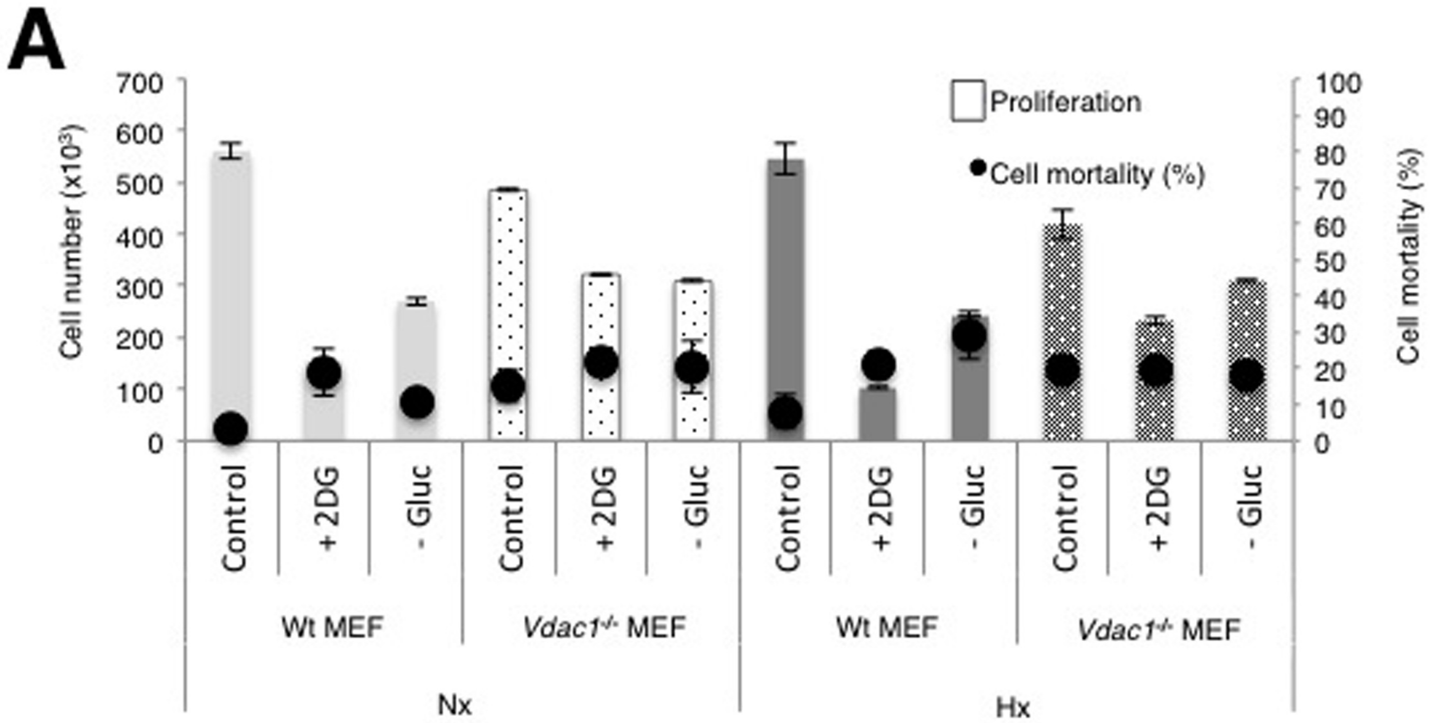

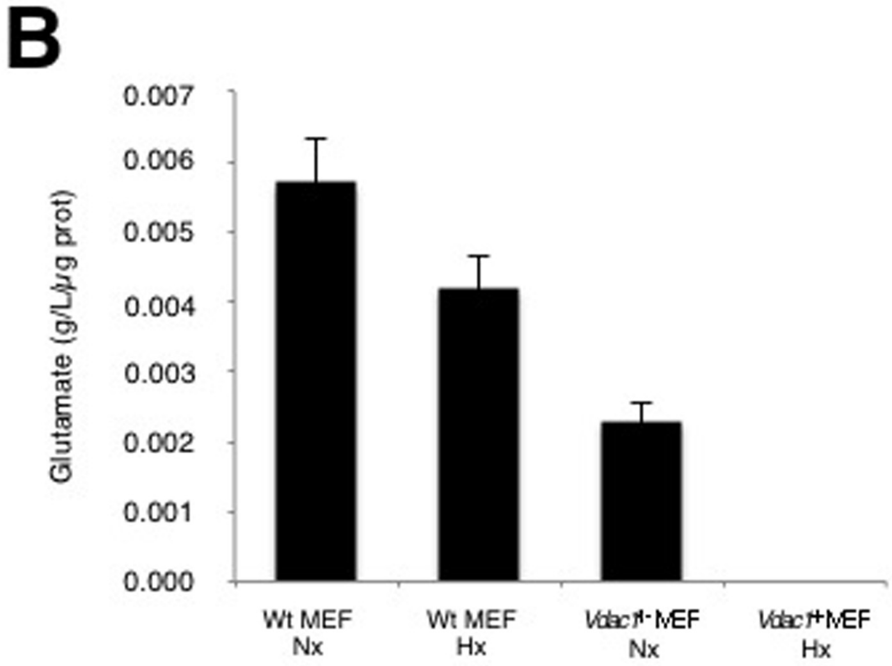

Supplemental Figure S7. Glucose and glutamine metabolism. (**A**) Wt and *Vdac1*$^{-/-}$ MEF were cultured for two days in Nx or Hx in the presence of 2-deoxy-D-glucose (2DG– 1µM) or without glucose (- Gluc). The cell number was measured on a Beckman Coulter apparatus (squares). The percentage of cell mortality was measured by trypan blue exclusion (black dots). The mean ±SEM is representative of two independent experiments carried out in duplicate. (**B**) The glutamate concentration in the media of Wt and *Vdac1*$^{-/-}$ MEF cultured in normoxia (Nx) or hypoxia 1% O_2 (Hx) was measured at day 7 using a YSI 2700 apparatus. *Vdac1*$^{-/-}$ MEF convert little or no glutamine into glutamate compared to Wt MEF.
