# Supplementary material for: Knockout of Vdac1 activates hypoxia-inducible factor through reactive oxygen species generation and induces tumor growth by promoting metabolic reprogramming and inflammation
Source: Cancer Metab. 2015 Aug 26;3:8. doi: 10.1186/s40170-015-0133-5 (PMC4551760; doi:10.1186/s40170-015-0133-5)
Supplement: Additional file 13: Figure S8. — Knockout of Vdac1 significantly disrupted apoptosis. Immunofluorescence to cytochrome C and DAPI in Wt and Vdac1 −/− MEF in Nx. [file 40170_2015_133_MOESM13_ESM.pdf]

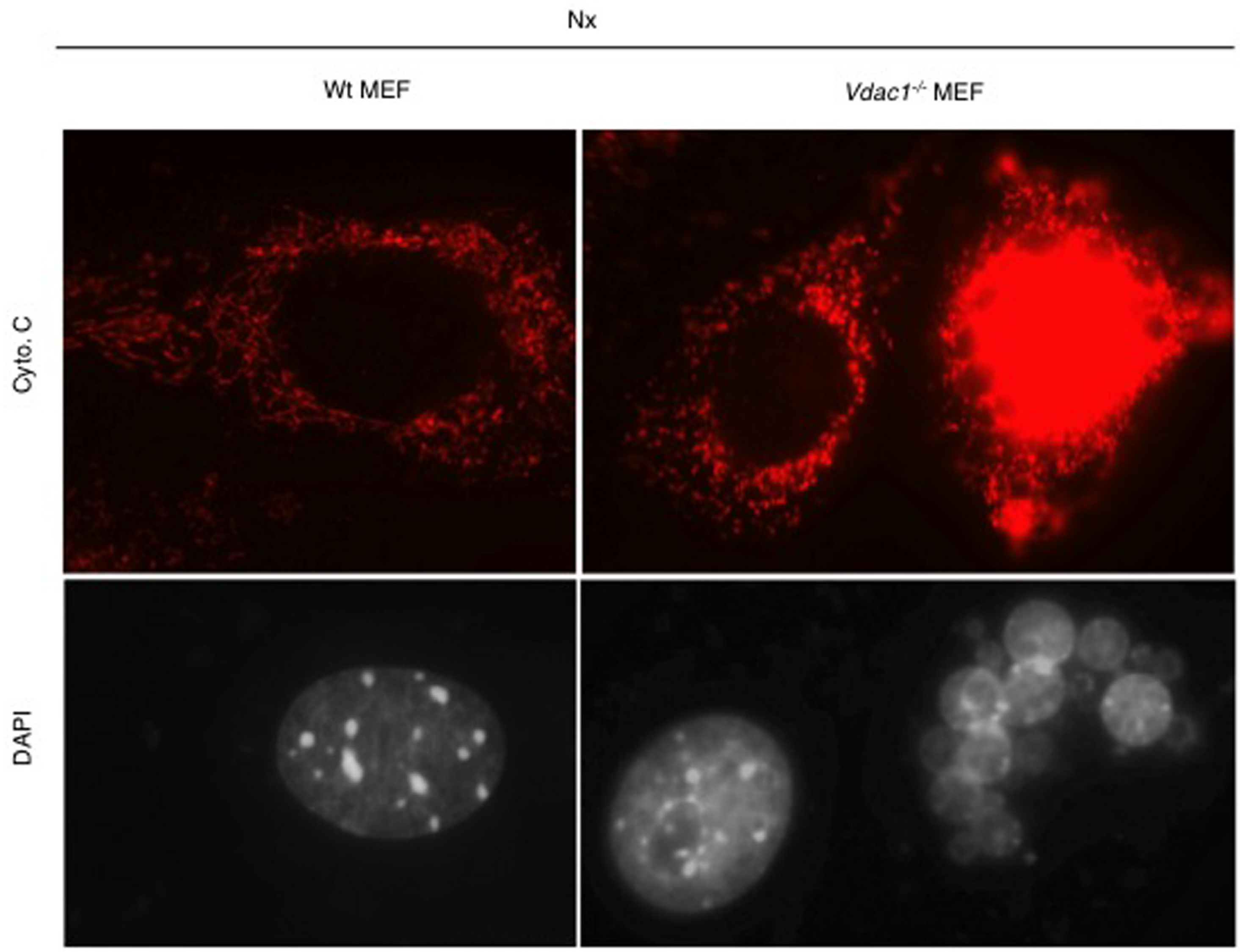

Supplementary Figure S8. Knockout of *Vdac1* significantly disrupted apoptosis. Immunofluorescence to cytochrome C and DAPI in Wt and *Vdac1*$^{-/-}$ MEF in Nx.
