# Supplementary material for: Knockout of Vdac1 activates hypoxia-inducible factor through reactive oxygen species generation and induces tumor growth by promoting metabolic reprogramming and inflammation
Source: Cancer Metab. 2015 Aug 26;3:8. doi: 10.1186/s40170-015-0133-5 (PMC4551760; doi:10.1186/s40170-015-0133-5)
Supplement: Additional file 14: Figure S9. — Autophagy was detected in Vdac1 −/− MEF. (A) Representative images of electron micrographs of Wt and Vdac1 −/− MEF incubated in normoxia (Nx) for 24 h. Vdac1 −/− MEF showed autophagosomes with double membranes (arrow). (B) Wt and Vdac1 −/− MEF lysates were analyzed in the absence (−) or presence of chloroquine (CQ) by immunoblotting to LC3. ARD1 was used as a loading control. The ratio of LC3-II/ARD1 was measured. [file 40170_2015_133_MOESM14_ESM.pdf]

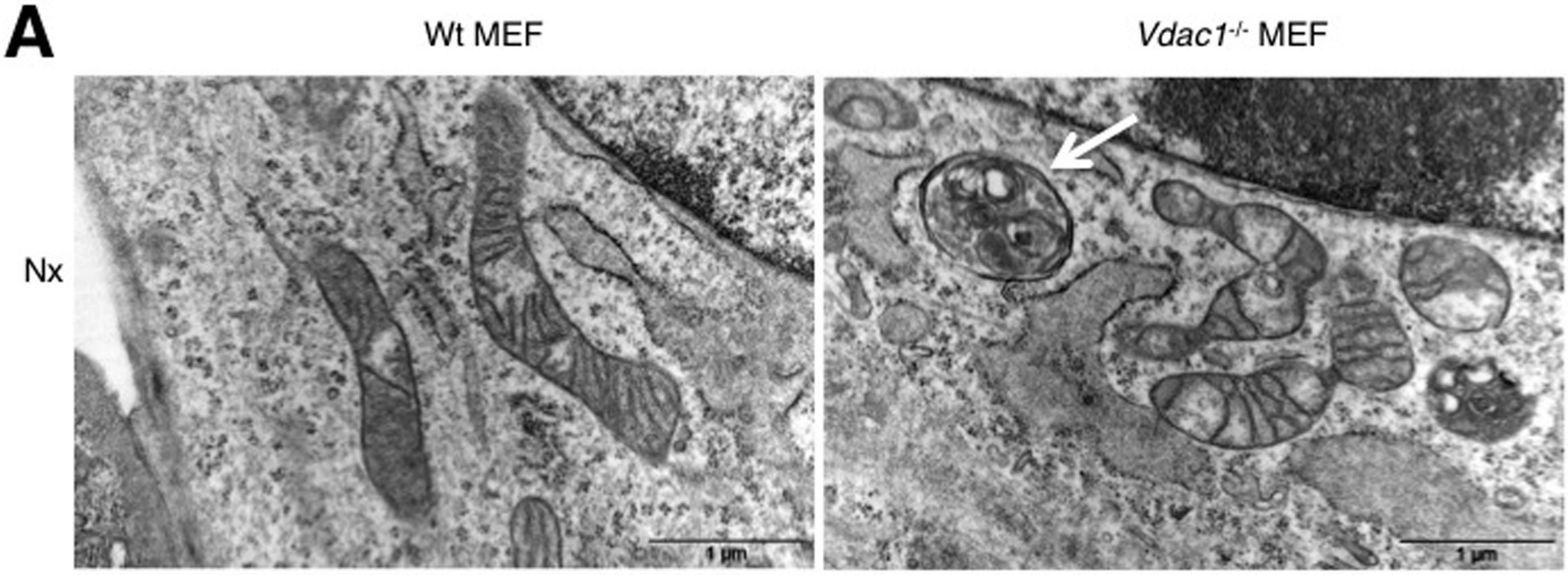

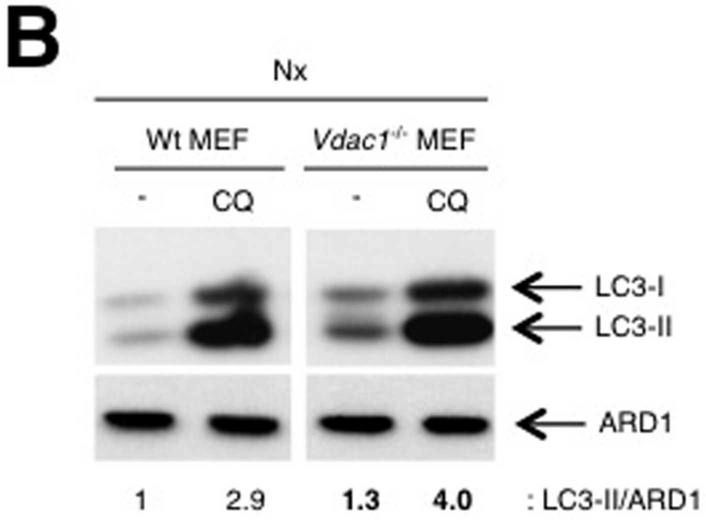

Supplemental Figure S9. Autophagy was detected in *Vdac1*$^{-/-}$ MEF. (A) Representative images of electron micrographs of Wt and *Vdac1*$^{-/-}$ MEF incubated in normoxia (Nx) for 24h. *Vdac1*$^{-/-}$ MEF showed autophagosomes with double membranes (arrow). (B) Wt and *Vdac1*$^{-/-}$ MEF lysates were analyzed in the absence (-) or presence of chloroquine (CQ) by immunoblotting to LC3. ARD1 was used as a loading control. The ratio of LC3-II/ARD1 was measured.
