# Supplementary material for: Knockout of Vdac1 activates hypoxia-inducible factor through reactive oxygen species generation and induces tumor growth by promoting metabolic reprogramming and inflammation
Source: Cancer Metab. 2015 Aug 26;3:8. doi: 10.1186/s40170-015-0133-5 (PMC4551760; doi:10.1186/s40170-015-0133-5)
Supplement: Additional file 15: Figure S11. — Red blood cells are present in Vdac1 −/− RAS MEF tumor tissue. (A) 20 mg of tumor tissue derived from Wt (1, 2, and 3) and Vdac1 −/− RAS MEF (4, 5, and 6) tumors were resuspended in RLT buffer before DNA/RNA/protein extraction. (B) Quantification of the number of blood vessels/microscopic field of CD31-positive vessels ± SEM of tumor tissue derived from Wt RAS (1, 2, and 3) and Vdac1 −/− RAS MEF (4, 5, and 6). (C) Average of the quantification of CD31-positive vessels ± SEM per microscopic field in Wt RAS MEF-derived tumors (Wt RAS MEF) and Vdac1 −/− RAS MEF-derived tumors (Vdac1 −/− RAS MEF). Statistical significance, p < 0.0001. (D) Immunofluorescence to hemoglobin (Hgb) in Vdac1 −/− RAS MEF. [file 40170_2015_133_MOESM15_ESM.pdf]

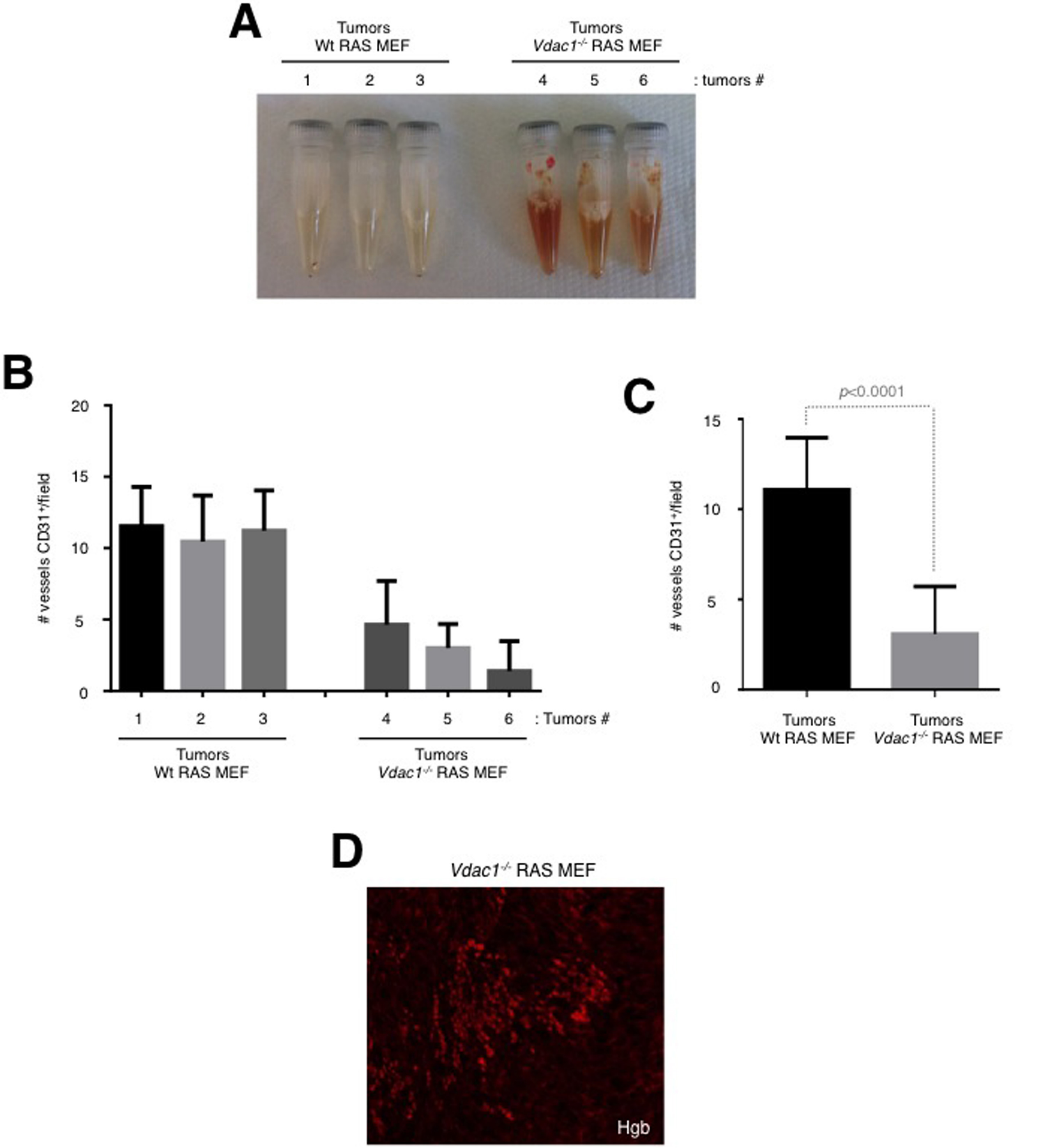

Supplemental Figure S11. Red blood cells are present in *Vdac1*$^{-/-}$ RAS MEF tumor tissue. (A) 20 mg of tumor tissue derived from Wt (1, 2 and 3) and *Vdac1*$^{-/-}$ RAS MEF (4, 5 and 6) tumors were resuspended in RLT buffer before DNA/RNA/protein extraction. (B) Quantification of the number of blood vessels/microscopic field of CD31-positive vessels ± SEM of tumor tissue derived from Wt RAS (1, 2 and 3) and *Vdac1*$^{-/-}$ RAS MEF (4, 5 and 6). (C) Average of the quantification of CD31-positive vessels ± SEM per microscopic field in Wt RAS MEF-derived tumors (Wt RAS MEF) and *Vdac1*$^{-/-}$ RAS MEF-derived tumors (*Vdac1*$^{-/-}$ RAS MEF). Statistical significance. $p<0.0001$. (D)
