# Supplementary material for: Knockout of Vdac1 activates hypoxia-inducible factor through reactive oxygen species generation and induces tumor growth by promoting metabolic reprogramming and inflammation
Source: Cancer Metab. 2015 Aug 26;3:8. doi: 10.1186/s40170-015-0133-5 (PMC4551760; doi:10.1186/s40170-015-0133-5)
Supplement: Additional file 16: Figure S12. — Immunohistochemical staining for VEGFA of tumor sections. (A) Negative control using secondary antibody only on Wt RAS MEFs. (B) VEGFA immunodetection in sections of Wt and Vdac1 −/− RAS MEF-derived tumor using 4X (top panels), 20X (middle panels), and 40X (bottom panels) magnification. [file 40170_2015_133_MOESM16_ESM.pdf]

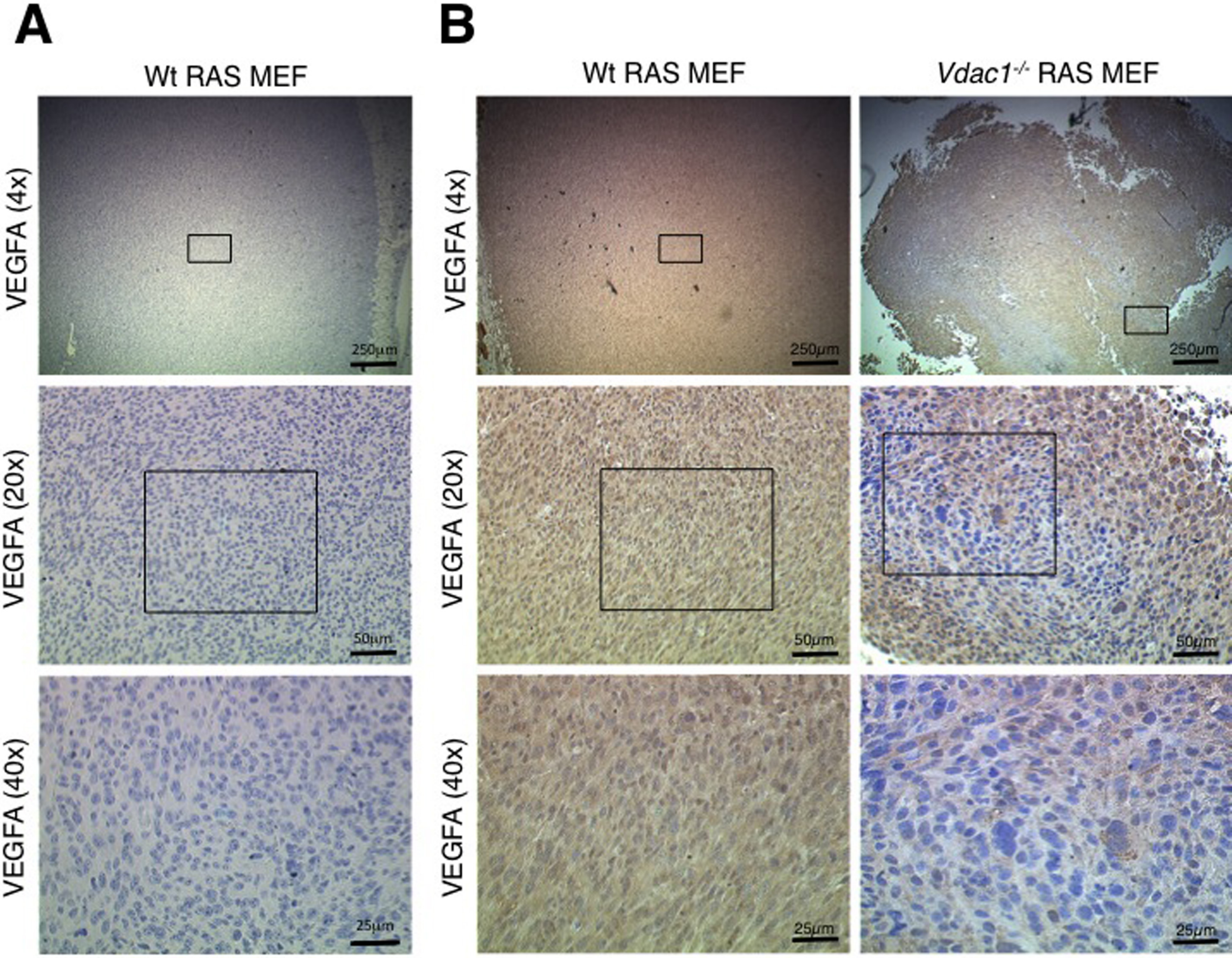

Supplemental Figure S12. Immunohistochemical staining for VEGFA of tumor sections. (A) Negative control using secondary antibody only on Wt RAS MEFs. **(B)** VEGFA immunodetection in sections of Wt and *Vdac1$^{-/-}$* RAS MEF-derived tumor using 4X (top panels), 20X (middle panels) and 40X (bottom panels) magnification.
