# Supplementary material for: Knockout of Vdac1 activates hypoxia-inducible factor through reactive oxygen species generation and induces tumor growth by promoting metabolic reprogramming and inflammation
Source: Cancer Metab. 2015 Aug 26;3:8. doi: 10.1186/s40170-015-0133-5 (PMC4551760; doi:10.1186/s40170-015-0133-5)
Supplement: Additional file 17: Figure S13. — VDAC1 expression in human tissues and cancers. (A) Heatmaps run from blue (low values, more dependency/essentiality) to white to red (high values, less dependency/essentiality). ATARiS gene-level data are median centered and therefore relative within the particular gene that is being viewed. (B) The Cancer Genome Atlas (TCGA) data sets of 89 cancer studies analyzed for mutation, deletion, gain, and amplification in the Vdac1 gene with cBioPortal. [file 40170_2015_133_MOESM17_ESM.pdf]

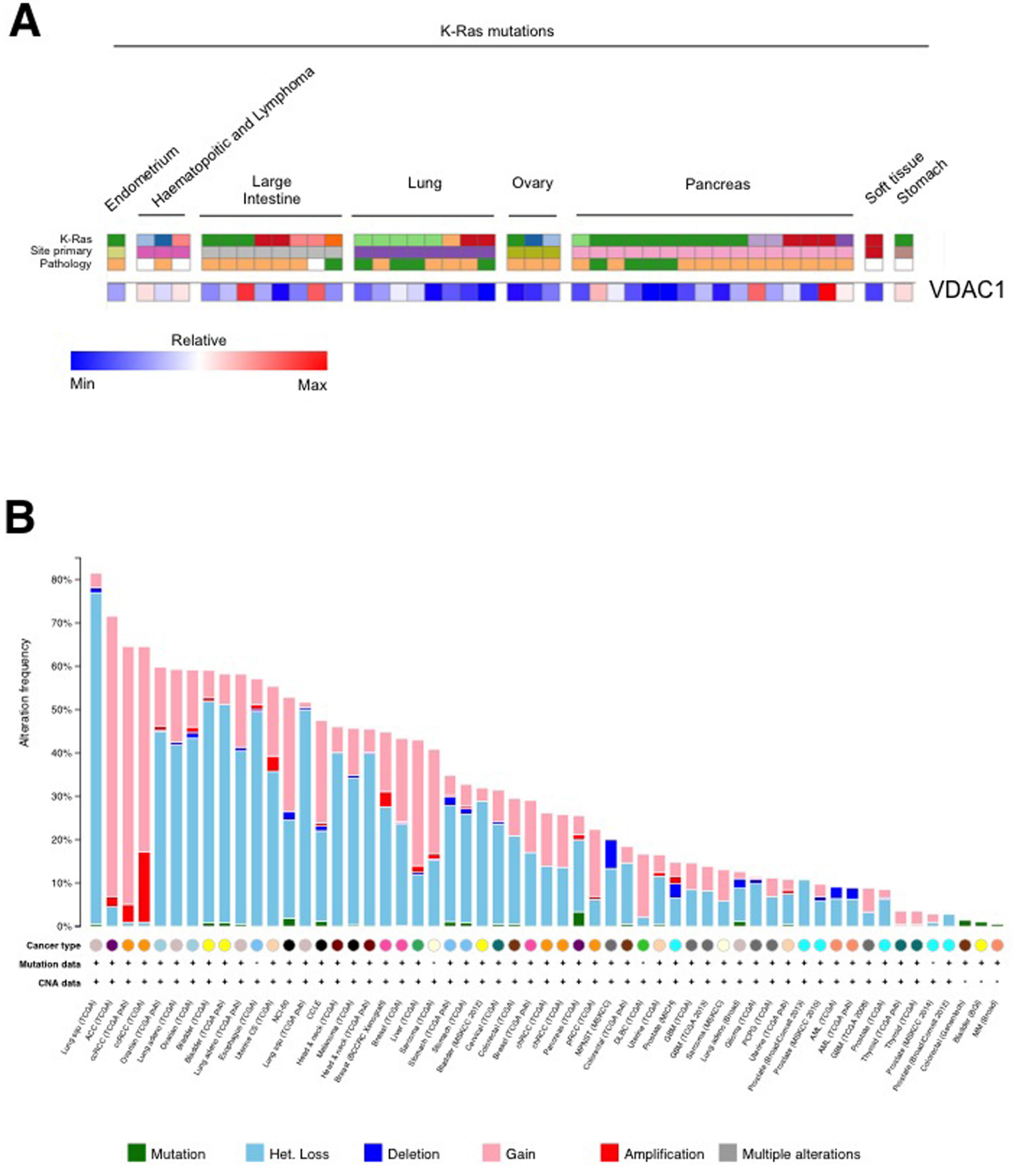

Supplemental Figure S13. VDAC1 expression in human tissues and cancers. (A) Heatmaps run from **blue** (low values, more dependency/ essentiality) to **white** to **red** (high values, less dependency/essentiality). ATARiS gene-level data are median centered and therefore relative within the particular gene that is being viewed. **(B)** The Cancer Genome Atlas (TCGA) data sets of 89 cancer studies analyzed for mutation, deletion, gain and amplification in the *Vdac1* gene with cBioPortal .
