# Supplementary material for: Knockout of Vdac1 activates hypoxia-inducible factor through reactive oxygen species generation and induces tumor growth by promoting metabolic reprogramming and inflammation
Source: Cancer Metab. 2015 Aug 26;3:8. doi: 10.1186/s40170-015-0133-5 (PMC4551760; doi:10.1186/s40170-015-0133-5)
Supplement: Additional file 18: Figure S10. — Characterization of RASV12 transformed MEF expressing (WT RAS MEF) or not (Vdac1 −/− RAS MEF) Vdac1. (A) Characterization of the growth of Wt and Vdac1 −/− RAS MEF incubated in Nx or Hx for the indicated number of days. The mean ± SEM is representative of four independent experiments carried out in duplicate. A p < 0.00001 shows significant difference from the normoxia for Vdac1 −/− RAS MEF. (B) Representative phase contrast photographs of Wt and Vdac1 −/− RAS MEF incubated in Nx for 72 h. Scale bars represent X μm. (C) Relative migration of Wt and Vdac1 −/− RAS MEF in Nx as evaluated in a xCELLigence system. The mean ± SEM is representative of two independent experiments carried out in quadruplicate. (D) The extracellular acidification rate (ECAR) in Nx of Wt and Vdac1 −/− RAS MEF was evaluated with a Seahorse XF bioenergetic system. Glucose (Glu 10 mM) and oligomycin (Oligo 1 μM) were injected at the indicated times. (E) The oxygen consumption rate (OCR) in Nx for Wt and Vdac1 −/− RAS MEF was measured in real time with a Seahorse XF. Glucose (Glu 10 mM), oligomycin (Oligo 1 μM), carbonilcyanide p-triflouromethoxyphenylhydrazone (FCCP 1 μM), and Rotenone/Antimycine A (Rot/AA, 1 μM/1 μM) were injected at the indicated times. The mean ± SEM is representative of three independent experiments carried out in quadruplicate. (F) Radioresistance of Wt and Vdac1 −/− RAS MEF cultured for 24 h in Nx or Hx and treated with the indicated dose of radiation. Cell growth was then evaluated with a clonogenic cell survival assay. X-axis: dose of X-radiation (Gy). Y-axis: surviving fraction. The mean ± SEM is representative of two independent experiments carried out in duplicate. (G) Soft agar assay of Wt and Vdac1 −/− RAS MEF. (H) Tumor weight of Wt (Wt RAS MEF) and Vdac1 −/− RAS MEF-derived tumors (Vdac1 −/− RAS MEF). [file 40170_2015_133_MOESM18_ESM.pdf]

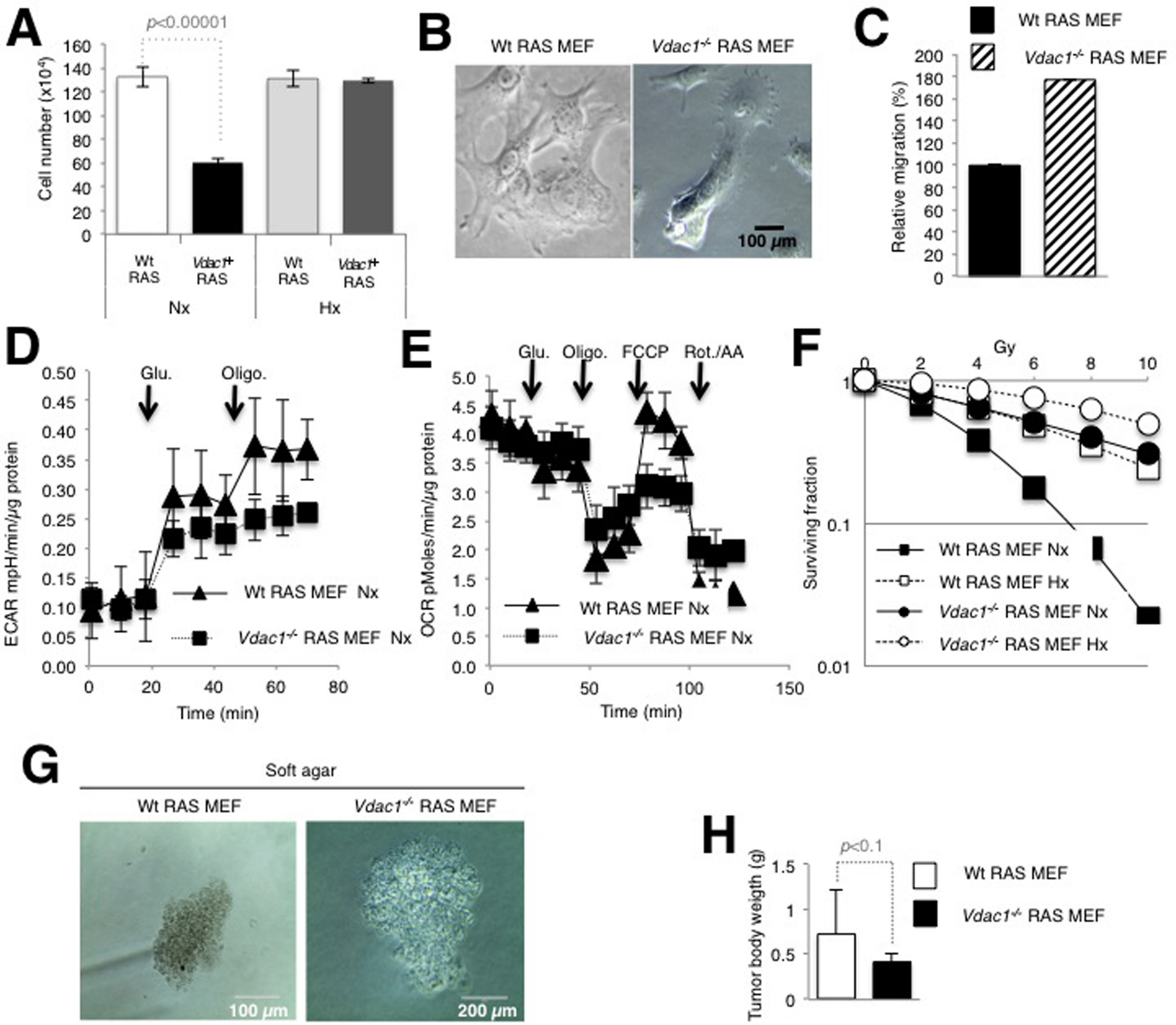

Supplemental Figure S10. Characterization of RASV12 transformed MEF expressing (WT RAS MEF) or not (*Vdac1*$^{-/-}$ RAS MEF) V*dac1*. (A) Characterization of the growth of Wt and *Vdac1*$^{-/-}$ RAS MEF incubated in Nx or Hx for the indicated number of days. The mean ±SEM is representative of four independent experiments carried out in duplicate. A $p<0.00001$ shows significant difference from the normoxia for *Vdac1*$^{-/-}$ RAS MEF. (B) Representative phase contrast photographs of Wt and *Vdac1*$^{-/-}$ RAS MEF incubated in Nx for 72h. Scale bars represent X µm. (C) Relative migration of Wt and *Vdac1*$^{-/-}$ RAS MEF in Nx as evaluated in a xCELLigence system. The mean ±SEM is representative of two independent experiments carried out in quadruplicate. (D) The extracellular acidification rate (ECAR) in Nx of Wt and *Vdac1*$^{-/-}$ RAS MEF was evaluated with a Seahorse XF bioenergetic system. Glucose (Glu 10mM) and oligomycin (Oligo 1µM) were injected at the indicated times. (E) The oxygen consumption rate (OCR) in Nx for Wt and *Vdac1*$^{-/-}$ RAS MEF was measured in real time with a Seahorse XF. Glucose (Glu 10 mM), oligomycin (Oligo 1µM), carbonilcyanide p-triflouromethoxyphenylhydrazone (FCCP 1 µM) and Rotenone/Antimycine A (Rot/AA, 1µM/1µM) were injected at the indicated times. The mean±SEM is representative of three independent experiments carried out in quadruplicate. (F) Radioresistance of Wt and *Vdac1*$^{-/-}$ RAS MEF cultured for 24h in Nx or Hx and treated with the indicated dose of radiation. Cell growth was then evaluated with a clonogenic cell survival assay. X-axis: dose of X-radiation (Gy). Y-axis: surviving fraction. The mean ±SEM is representative of two independent experiments carried out in duplicate. (G) Soft agar assay of Wt and *Vdac1*$^{-/-}$ RAS MEF. (H) Tumor weight of Wt (Wt RAS MEF) and *Vdac1*$^{-/-}$ RAS MEF-derived tumors (*Vdac1*$^{-/-}$ RAS MEF).
